# Lipid-Modulated, Graduated Inhibition of N-Glycosylation Pathway Priming Suggests Wide Tolerance of ER Proteostasis to Stress

**DOI:** 10.1021/acscentsci.4c01506

**Published:** 2024-12-26

**Authors:** Andrew M. Giltrap, Niamh Morris, Yin Yao Dong, Stephen A. Cochrane, Thomas Krulle, Steven Hoekman, Martin Semmelroth, Carina Wollnik, Timea Palmai-Pallag, Elisabeth P. Carpenter, Jonathan Hollick, Alastair Parkes, York Rudhard, Benjamin G. Davis

**Affiliations:** † The Rosalind Franklin Institute, Harwell Science & Innovation Campus, Harwell OX11 0FA, U.K.; ¶ MRC Weatherall Institute of Molecular Medicine, John Radcliffe Hospital, Oxford OX3 9DS, U.K.; § Department of Chemistry, University of Oxford, Oxford OX1 3TA, U.K.; ⊥ Evotec, 114 Innovation Drive, Milton Park, Abingdon, Oxfordshire OX14 4RZ, U.K.; # Evotec SE, Manfred Eigen Campus, Essener Bogen 7, 22419 Hamburg, Germany; ∇ Structural Genomics Consortium, University of Oxford, Oxford OX3 7DQ, U.K.; ○ Department of Pharmacology, University of Oxford, Oxford OX1 3QT, U.K.; ● Department of Chemistry, University of Technology Sydney, 15 Broadway, Ultimo, Sydney, NSW 2007, Australia

## Abstract

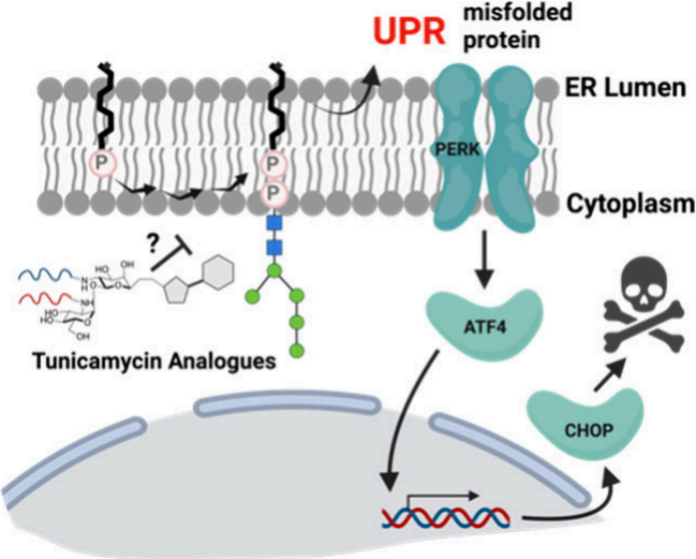

Protein N-glycosylation is a cotranslational modification that takes place in the endoplasmic reticulum (ER). Disruption of this process can result in accumulation of misfolded proteins, known as ER stress. In response, the unfolded protein response (UPR) restores proteostasis or responds by controlling cellular fate, including increased expression of activating transcription factor 4 (ATF4) that can lead to apoptosis. The ability to control and manipulate such a stress pathway could find use in relevant therapeutic areas, such as in treating cancerous states in which the native ER stress response is often already perturbed. The first committed step in the N-glycosylation pathway is therefore a target for potential ER stress modulation. Here, using structure-based design, the scaffold of the natural product tunicamycin allows construction of a panel capable of graduated inhibition of DPAGT1 through lipid-substituent-modulated interaction. The development of a quantitative, high-content, cellular immunofluorescence assay allowed precise determination of downstream mechanistic consequences (through the nuclear localization of key proxy transcription factor ATF4 as a readout of resulting ER stress). Only the most potent inhibition of DPAGT1 generates an ER stress response. This suggests that even low-level “background” biosynthetic flux toward protein glycosylation is sufficient to prevent response to ER stress. “Tuned” inhibitors of DPAGT1 also now seemingly successfully decouple protein glycosylation from apoptotic response to ER stress, thereby potentially allowing access to cellular states that operate at the extremes of normal ER stress.

## Abstract 2

Editing of the archetypal tunicamycin scaffold creates novel intracellular protein glycosylation inhibitors with altered stress induction potential.

## Introduction

The endoplasmic reticulum (ER) is the subcellular compartment responsible for N-linked glycosylation of proteins. This cotranslational modification is critical for ensuring the proper folding, stability, and localization of many proteins.^1^ Indeed, genetic “knockouts” of this pathway are typically embryonically lethal, and mutations in any of the enzymes in this complex pathway result in severe congenital diseases of glycosylation.^2^

**Figure 1:**
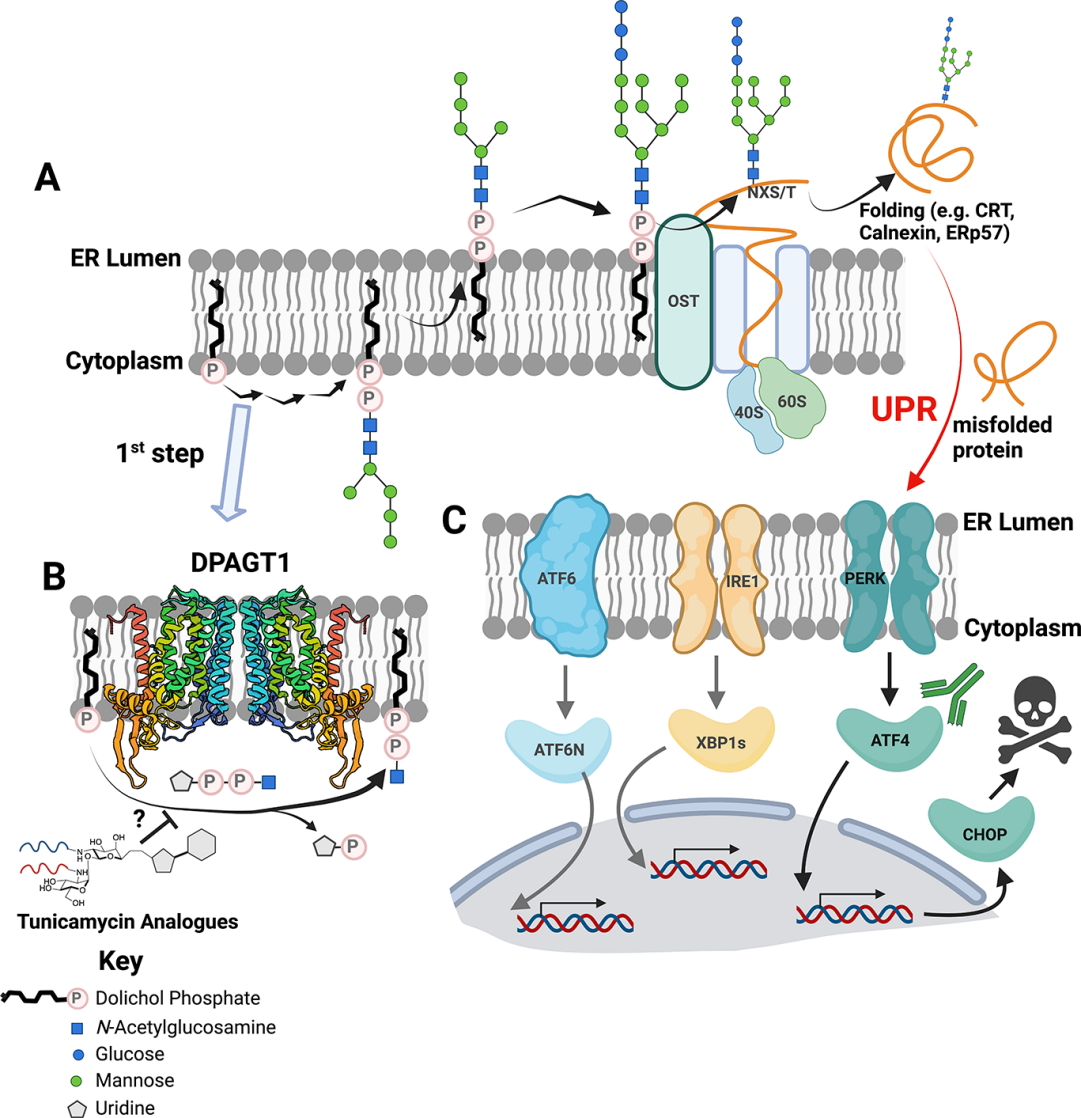
**The mammalian protein N-glycosylation pathway in the endoplasmic reticulum as a putative driver of cellular stress.** (A) Dolichol phosphate (black) is elaborated via numerous enzymes to generate a heptasaccharide unit before being flipped to the ER lumen and further elaborated. The mature glycan is transferred to proteins by OST and folded. (B) The first step in this N-glycosylation pathway is the transfer of phospho-GlcNAc to dolichol phosphate catalyzed by DPAGT1, thereby providing a putative target for pharmacological induction of stress via inhibition. (C) Accumulation of misfolded proteins triggers the UPR via signaling at one of three receptors. Activation of PERK leads to generation of the transcription factor ATF4 and subsequent expression of CHOP, which in severe UPR leads to cell death. Created in BioRender. https://BioRender.com/s20z883

Perturbation of the N-glycosylation pathways in the ER (Figure 1A) leads to the accumulation of mis- or unfolded proteins and results in a cellular phenomenon known as ER stress. To maintain normal function, the cell initiates the unfolded protein response (UPR) to restore homeostasis (Figure 1). Three transmembrane proteins in the ER act as sensors for ER stress: activating transcription factor 6 (ATF6),^3^ inositol-requiring enzyme 1 (IRE1),^4^ and PKR-like ER kinase (PERK).^5^ In the presence of unfolded proteins in the ER, these sensors can signal, thereby increasing expression of chaperones for protein folding, protein degradation, and translational inhibition to limit protein generation.^6^ However, if normal functioning is not restored by the UPR, sustained activation of PERK results in increased expression of activating transcription factor 4 (ATF4)^7^ and subsequent generation of the pro-apoptotic transcription factor CCAAT-enhancer-binding protein homologous protein (CHOP), which leads ultimately to cell death via apoptosis (Figure 1C).^8^

ER stress modifiers, and manipulators of the UPR, may therefore have profound effects on cell survival or as treatments, such as in anticancer regimes,^9−11^ that may exhibit selective killing. Such modifiers have also been suggested but not proven as possible therapeutic approaches for a number of other diseases areas, including neurodegeneration^12^ and immune modulation.^13^ However, due to the multifaceted nature of the UPR, there remains a lack of specific and tunable small-molecule modulators of this pathway to better understand mechanism. Moreover, since modulation of kinase PERK^14^ has broadscale systematic effects (e.g., pancreatic toxicity^15^) and since ATF4 and CHOP^16^ are downstream transcription factors, their direct targeting in the downstream PERK–ATF4–CHOP pathway is not readily tractable by many current strategies.

As a primary determinant for correctly folded protein, N-glycosylation is therefore potentially one of the most potent upstream “levers” for indirect control of this pathway, yet the exploration of analogues and consequent mechanisms that may modulate this pathway for an effect upon ER stress has not to our knowledge been previously studied. Here, through the development of a coherent cellular interrogation strategy, we link systematic variation based on structural analyses to create a panel of graduated inhibitors of the N-linked glycosylation pathway. When coupled with the use of quantitative, high-content microscopy, this allows precise dissection of downstream transcription factor localization and hence activation in larger cellular populations. This reveals not only that ER stress is induced only at extremes of reduced flux (and hence is a robust pathway) but also that there is a clear dose–response “window” before onset of observed cytotoxicity. Together these data suggest that N-glycosylation may now be successfully uncoupled, at least in part, from ER stress responses that in turn may potentially be uncoupled from cell death.

## Results

### Design of a Strategy for Exploiting N-Glycosylation Pathway-Induced Stress

Despite the complexity of the N-glycosylation pathway, which utilizes multiple sequential enzymes, each responsible for the transfer of a given sugar to produce the mature glycan that is attached to a protein (Figure 1A), the first committed step provides a potentially universal control point. In mammals, this is catalyzed by the enzyme DPAGT1, a member of the polyisoprenyl-phosphate *N*-acetylaminosugar-1-phosphoryl transferases (PNPTs).^17^ This class of glycan-processing enzymes catalyzes the transfer of glycosyl phosphates, here to the lipid carrier dolichol phosphate, via the formation of a pyrophosphate linkage (Figure 1B).^18^

**Figure 2:**
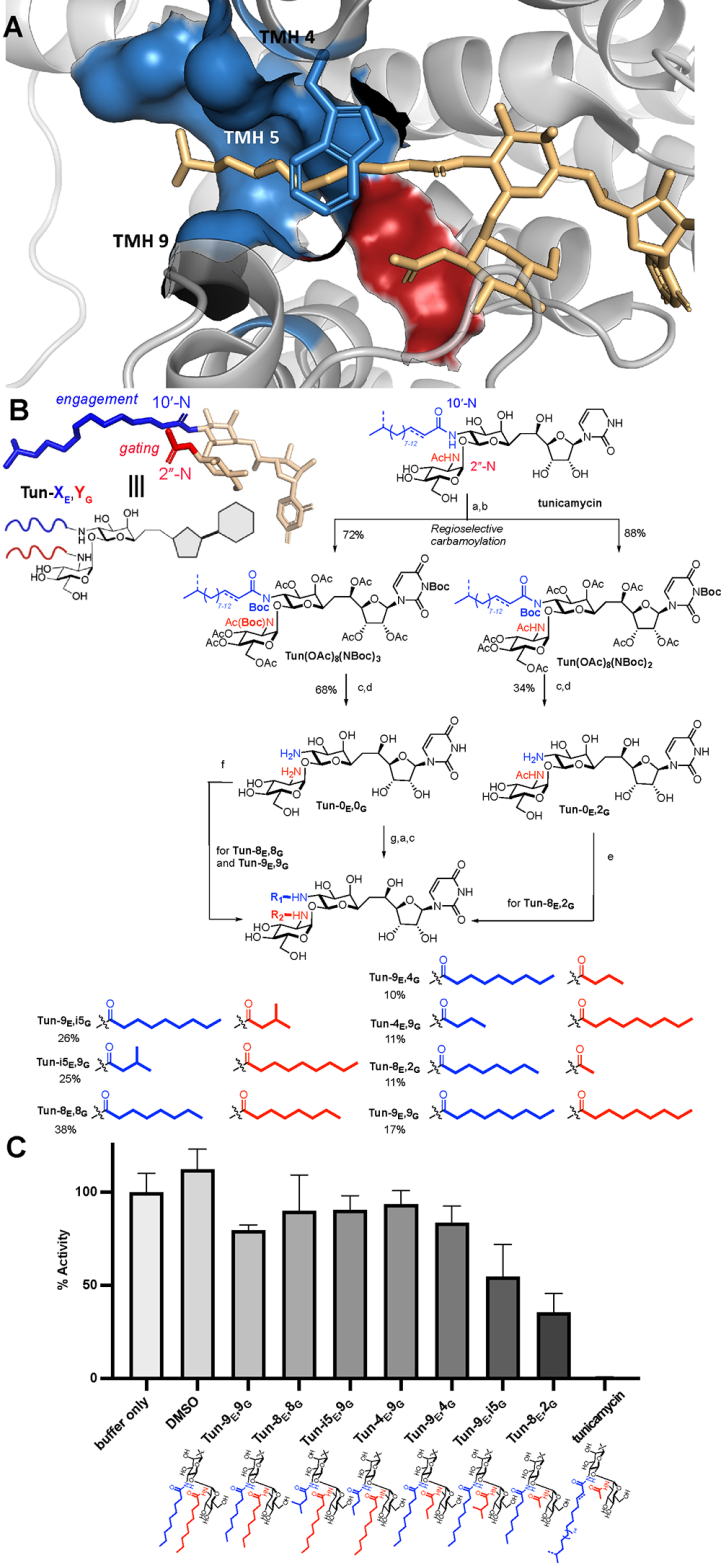
**Design, synthesis and *in vitro* inhibitory activity of lipid-altered tunicamycins.** (A) Structure of DPAGT1 in complex with tunicamycin^21^ showing key interactions primarily along TMH5 that in concert with a Trp122 “lid” create the lipid-engagement site (blue). This engagement contrasts with a small, gated pocket below bounded primarily by Leu293 in which only smaller moieties (here an acetamide) are accommodated (red). (B) Guided by this structure, as an apparent snapshot of a Michaelis complex-like mode of binding, modulation at two acyl sites in the core scaffold was explored. This systematically altered lipid engagement (E) substituents **X**_**E**_ (blue) at 10′-N and gating (G) substituents **Y**_**G**_ (red) at 2″-N as key putative sites of differentiation in analogues **Tun-X**_**E**_**,Y**_**G**_, intended to synergistically modulate binding to DPAGT1 and hence create graduated inhibition. Semisynthesis allowed generation of graduated lipid tunicamycin analogues through regiochemical differentiation in carbamoylation to generate two key divergent intermediates: **Tun-0**_**E**_**,0**_**G**_ and **Tun-0**_**E**_**,2**_**G**_. Subsequent differential acylations allow for generation of both symmetric and asymmetric **Tun-X**_**E**_**,Y**_**G**_ analogues. Reagents and conditions: (a) Ac_2_O, pyridine; (b) Boc_2_O, DMAP, THF; (c) NaOMe, MeOH; (d) 4 M HCl in dioxane, MeOH; (e) HATU, DIPEA, octanoic acid, DMF; (f) HATU, DIPEA, carboxylic acid; (g) HATU, DIPEA, 2 × carboxylic acid. (C) Residual % *in vitro* activity of DPAGT1 following incubation with analogues [DPAGT1 (1 mM), MgCl_2_ (5 mM), Tun analogue (1 mM), dolichol phosphate (50 mM), and UDP-GlcNAc(1-^14^C) (50 mM) in 1% OGNG/CHS/cardiolipin] was assessed through end point assay at 37 °C via phosphorimaging. These were determined on the basis of three biological replicates, each using three technical replicates. Error bars shown are standard deviations.

Tunicamycin (Figure 2), a natural product isolated from *Streptomyces* sp., is a rare inhibitor of PNPTs. It can, for example, be utilized to inhibit MraY, a key PNPT enzyme at the beginning of bacterial cell wall biosynthesis,^19^ and this has sparked interest as a potential antibiotic lead. However, its canonical role as an inhibitor of the eukaryotic N-glycosylation pathway through action upon PNPT DPAGT1 suggested it as a potential modulator of mammalian ER stress through this mechanism.^20^

Recently, we reported the structure-based, rational design of PNPT-selective compounds.^21^ In this way we created, for example, nontoxic tunicamycin analogues that displayed selective inhibitory activity against *bacterial* PNPT MraY but did not inhibit *mammalian* PNPT DPAGT1. Based on this demonstration of a clean “on–off” distinction between inhibition profiles of PNPT activity via the mechanistic and structural analysis of DPAGT1, we also considered that graduated activity targeted instead toward human PNPT DPAGT1 might also be designed to give putative probes of ER stress and, as a result, possible associated thresholds for control of downstream eukaryotic cellular function.

The structure of DPAGT1 in complex with the archetypal scaffold natural product tunicamycin^21^ (Figure 2A) reveals a key concave groove formed along transmembrane helix 5 (TMH5) and between helices TMH4 and TMH9 that is occupied by tunicamycin’s longer fatty acid chain (from the 10′-N position). First, this suggested that it is this substituent at position 10′ in tunicamycin that is capable of partially mimicking the C_∼100_ chain of DPAGT1’s substrate dolichol-1-phosphate by engaging with this pocket. Trp122 moves so as to use its indole moiety to hydrophobically provide a binding “cap” or “lid” over the proximal portion of the 10′-N lipid chain. This in turn suggested that a minimal-length lipid would be required at this position in any probe compounds to interact with this lipid-binding tunnel and “cap/lid”—a key lipid binding site (blue, Figure 2A)—and hence to target DPAGT1. The strength of this interaction in contributing to this Michaelis complex-like mimicry is supported by the striking stabilization of DPAGT1 stability toward denaturation in heat-induced denaturation assays when bound to tunicamycin (*T*_m,1/2_ = 83.0 ± 0.4 °C for DPAGT1·tunicamycin vs 51.7 ± 0.2 °C for DPAGT1; Δ*T*_m,1/2_ = 31.3 °C), which is much greater than that provided by the Dol-1-P substrate (*T*_m,1/2_ = 58.5 ± 0.3 °C for DPAGT1·Dol-1-P; Δ*T*_m,1/2_ = 5.5 °C).

Second and importantly, we reasoned that the other TMH-9-directed face of TMH5 of DPAGT1 displays residues that progressively “gate” the 2″-N substituent of tunicamycin (2″-N acetamide in the wild-type natural product, red in Figure 2A). We thus reasoned these two sites—the lipid engagement site (10′-N, blue) and the gating site (2″-N, red)—might allow tuning of binding and hence DPAGT1 inhibition activity through careful manipulation of the size of lipid substituents in glycomimetics that interact at these two sites. In this way, tensioning of positive lipid engagement (blue, Figure 2A,B) versus negative lipid gating (red, Figure 2A,B) would thus probe a designed “biting point” between inhibition of protein glycosylation and the onset of cellular ER stress.

### Synthesis of Potentially Graduated ER-Stress Inducers Based upon the Tunicamycin Core Scaffold

We therefore designed a strategy to install graduated “engaging” and “gating” lipids at the 10′-N and 2″-N positions, respectively (Figure 2B), via chemoselective amidation of suitably deprotected polyhydroxylated scaffolds. In this way, the tunicamycin core scaffold bears two sites that could be independently addressed in principle and therefore varied. Our construction strategy relied on a gram-scale semisynthetic approach starting from tunicamycin itself, initially beginning with peracetylation to generate **Tun(OAc)**_**8**_. Complete imidation using di-*tert*-butyl dicarbonate allowed installation of Boc groups at both the 2″-N and 10′-N sites as well as at the 3-N of the uracil moiety, affording **Tun(OAc)**_**8**_**(NBoc)**_**3**_. Subsequent mild basic alcoholysis with sodium methoxide solution then allowed selective cleavage of the native acyl chains^22^ at both 2″-N and 10′-N as well as concomitant global deacetylation to generate the tris(carbamate) **Tun(NBoc)**_**3**_. Subsequent cleavage of the Boc groups with mild acid then generated the key bis(amine) intermediate **Tun(NH**_**2**_**)**_**2**_ bearing free amino groups at the lipid gating (2″-N) and engagement (10′-N) sites, primed for modification (Figure 2B), also named here **Tun-0**_**E**_**,0**_**G**_ (where ***n*_E_** and ***n*_G_** represent the *C*-acyl chain lengths installed at the engagement and gating sites, respectively).

Notably, tuning of carbamate formation revealed reduced nucleophilicity of the 2″-N amide in the α-glucosaminide moiety, consistent with its presence in a *gauche*,*cis*-1,2-glycoside. This therefore also valuably allowed access to a 10′-*N*,3-*N*-di-Boc bis(carbamate) variant (**Tun(OAc)**_**8**_**(NBoc)**_**2**_). Following alcoholytic 10′-N deacylation and global deprotection, this therefore allowed access to the monoamine, named here **Tun-0**_**E**_**,2**_**G**_.

These unprotected amino scaffolds, **Tun-0**_**E**_**,0**_**G**_ and **Tun-0**_**E**_**,2**_**G**_, proved to be versatile intermediates for diversification (Figure 2B). First, symmetric dual-site analogues were readily accessed through direct uronate-mediated amide coupling of bis(amine) **Tun-0**_**E**_**,0**_**G**_ to generate bislipidated compounds **Tun-8**_**E**_**,8**_**G**_ and **Tun-9**_**E**_**,9**_**G**_ carrying eight-carbon bis(octanoyl) and nine-carbon bis(nonanoyl) 10′-N and 2″-N substituents, respectively. Second, similar use of monoamine **Tun-0**_**E**_**,2**_**G**_ under essentially identical conditions gave direct access to the asymmetric variant **Tun-8**_**E**_**,2**_**G**_ bearing an eight-carbon chain at the engagement site 10′-N and yet a two-carbon moiety at the gating site 2″-N. Third, due to the valuably different physical properties of such asymmetric variants, a statistically distributive coupling procedure proved to be possible with bis(amine) **Tun-0**_**E**_**,0**_**G**_. Thus, coincident treatment with fatty acid “pairs” in equimolar amounts in the presence of uronate allowed ready access (following peracetylation–isolation–deacetylation) to additional desired asymmetric, site-varied analogues **Tun-9**_**E**_**,4**_**G**_ and **Tun-9**_**E**_**,i5**_**G**_ and their “site-reversed” counterparts **Tun-4**_**E**_**,9**_**G**_ and **Tun-i5**_**E**_**,9**_**G**_. In this way, a systematic panel of analogues was created that would allow graduated probing of both “engagement” (via 10-N′) and “gating” (via 2-N″) using long (eight/nine-carbon), medium (four-carbon), branched (*iso*-five-carbon, *i5*) and short (two-carbon) substituents off a common glycomimetic core natural product scaffold.

### Delineation of Putative PNPT Potency

The intended targeting and graduated modulation of DPAGT1’s PNPT activity was assessed *in vitro* using a highly pure recapitulated source. Briefly, full-length DPAGT1 bearing an additional tobacco etch virus (TEV) protease-cleavable N-terminal His_6_ tag was expressed using a baculovirus (DH10Bac)/insect cell (*Spodoptera frugiperda* Sf9) system using a pFB-LIC-Bse expression vector. DPAGT1 is an integral transmembrane enzyme, and thus, after harvesting and lysis, protein was extracted from the expression host membrane fraction using octyl glucose neopentyl glycol (OGNG) (1% w/v) and cholesterylhemisuccinate (CHS) (0.1% w/v) before isolation and purification by immobilized metal affinity chromatography (IMAC) using a Co^2+^-charged (“TALON”) resin and elution with an imidazole gradient. To ensure that there were no confounding effects of the His_6_ tag, this was then cleaved with TEV protease followed by reverse IMAC and size exclusion chromatography in sequence.

Subsequent three-component, mixed-phase CHS/OGNG/cardiolipin buffered *in vitro* assay revealed this as an active, highly stable protein source. Radiometric bisubstrate analyses allowed excellent sensitivity under the conditions necessary for proper recapitulation of the activity. Purified DPAGT1 (1 μM) was incubated with both substrates, dolichol monophosphate and radiolabeled UDP-*N*-acetyl-d-[1-^14^C]glucosamine, at 50 μM, and the activity was determined directly by end point phosphor detection of product dolichol-PP-*N*-acetyl-d-[1-^14^C]glucosamine formation. Putative inhibitors were incubated at a concentration of 1 μM, equivalent to an enzyme:inhibitor testing ratio of 1:1, representative of estimated local membrane concentrations and hence excess of substrate (∼50-fold) found in an intracellular context.^23,24^

Pleasingly, consistent with design, a clear tuning of inhibition was observed for different lipid-modulated analogues with altered engagement and gating (Figure 2C). Thus, “large” (>8-carbon) lipid at the gating site (red) resulted in little to no significant inhibition of the enzyme (**Tun-9**_**E**_**,9**_**G**_, **Tun-i5**_**E**_**,9**_**G**_, **Tun-4**_**E**_**,9**_**G**_, **Tun-8**_**E**_**,8**_**G**_). Reduction of the gating substituent size to branched (**Tun-9**_**E**_**,i5**_**G**_) and then short (**Tun-8**_**E**_**,2**_**G**_) variants generated increasing inhibitory activity in a graduated manner and hence modulated reduction in DPAGT1 activity as intended. Extension of the engagement site lipid length (to 12 to 16 carbons, in tunicamycin) drove complete inhibition,^21^ thereby further highlighting the dual opposing contributions from substituents at 10′-N and 2″-N. Interestingly, medium-sized gating (**Tun-9**_**E**_**,4**_**G**_) did not cause a significant reduction in DPAGT1 activity, and this may reflect previously inferred^25−28^ conformational flexibility of *n*-butanoyl amide moieties within ligands that can result in inconsistent or flexible pocket engagement. In this way, tuning the size of the lipid substituents at both so-called engagement (blue) and gating (red) sites resulted in clear modulation of DPAGT1 activity, generating inhibitors which range from zero to partial to full inhibition under these conditions. We next sought to investigate the effect of such attenuated DPAGT1 activity on the downstream protein glycosylation pathway and the subsequent onset of ER stress and its consequences.

### Minimal Protein Glycosylation Flux Is Enough to Prevent the ER Stress Response

Our intended manipulation of the ER stress response via blocking of a step (catalyzed by DPAGT1) that is a highly “upstream” point in a pathway critical to the biosynthesis of folded proteins required a suitable “downstream” proxy of relevant response. The quantitative and statistically significant measurement of such relevant proxy signals in biosynthetic pathways to unpack mechanism is a general and open question in cell biology.^29^ Our successful graduated inhibition of DPAGT1 at this critical flux point in the protein glycosylation pathway suggested the potential of the lipid-modulated tunicamycins in a relevant cellular system (here human embryonic kidney (HEK) cells) in which both protein glycosylation^30^ and ER stress^31^ have been shown to be representative of broader physiological function. ATF4 can be considered the direct signaling precursor to subsequent expression of CHOP and then cell death via apoptosis. As the critical transcription factor for this event, we therefore considered that its relocalization from cytoplasm to nucleus would provide a clear, unambiguous, and potentially high-content signal at a significantly downstream point yet possibly with different dose–response behaviors to events upstream (e.g., DPAGT1 inhibition) or downstream (e.g., cytotoxicity). In this way, this distant connection of an “upstream” intervention to “downstream” functional proxy allowed potentially global assessment of intervening cellular response and identification of windows of differential response tool molecules.

**Figure 3:**
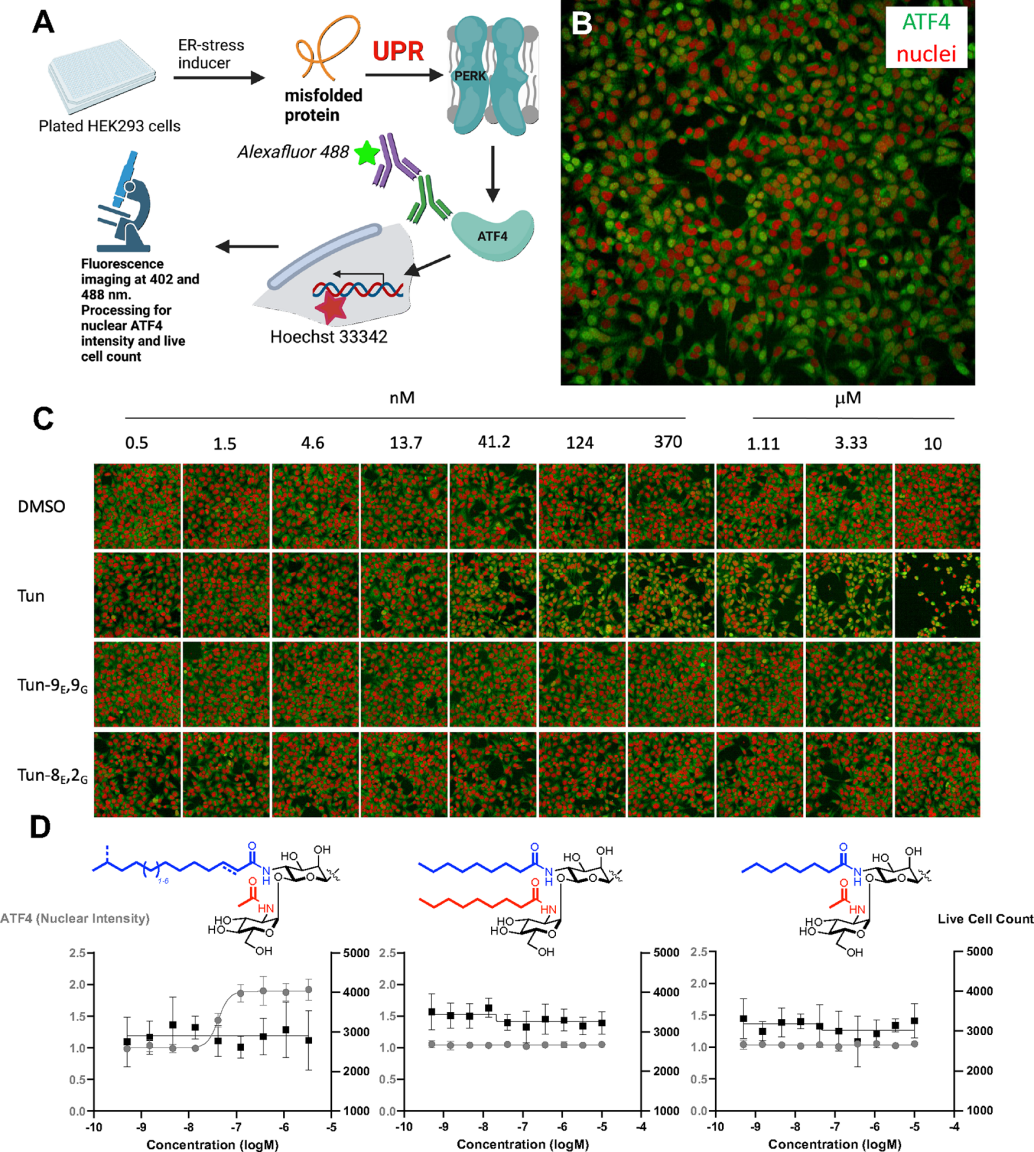
**High-content phenotypic screen for putative ER stress inducers.** (A) Schematic of the screening process. 12,000 HEK293 WT cells per well were plated into a 384-well plate and incubated at 37 °C for 24 h. Compounds were serially diluted and added to the plate and incubated for 6 h at 37 °C, at which point the cells were fixed with 4% PFA in PBS. The plates were then submitted to indirect ATF4 immunofluorescence, in which the plate was incubated with a primary anti-ATF4 (D4B8) antibody, followed by Alexa Fluor 488-labeled goat anti-rabbit secondary antibody and Hoechst 33342 dye. Plates were imaged using an OPERA-Phenix high-content imaging platform with fluorescence excitation at 405 and 488 nm. The images were analyzed, and the nuclear intensity of ATF4 for each cell was quantitatively determined (gray filled circles, left-hand axis in (D)) as well as the count of live cells (black squares, right-hand axis in (D)). Created in BioRender. https://BioRender.com/z32w873 (B) Representative section of imaging (red = nuclei, green = ATF4) for a given cellular population at a single dose (here exposed to tunicamycin at 41.2 nM). (C) Representative series panels of dose–response images after exposure to three different **Tun-X**_**E**_**,Y**_**G**_ analogues and DMSO control. (D) Corresponding resulting quantification of the high-throughput microscopy assay displaying the live cell count (black, right axis) and the nuclear intensity of ATF4 (gray, left axis) of tunicamycin and two lipid altered analogues, one with a large lipid at the gating position (**Tun-9**_**E**_**,9**_**G**_) and other with a short lipid at this position (**Tun-8**_**E**_**,2**_**G**_). See the Supporting Information for full details.

Here, the nuclear localization of the transcription factor ATF4 (which leads to subsequent expression of CHOP and cell death) was used as a phenotypic marker of ER stress (Figure 3A). Use of multi(384)-well plate format allowed wild-type HEK cells to be treated with analogues over a titrating concentration range of 0.5 nM to 10 mM for 6 h in a quantitative manner (Figure 3C). After fixation with paraformaldehyde, high-content cellular fluorescence microscopy imaging was used to both determine the site and quantify the location of ATF4^32^ (Figure 3B,C). Thus, immunofluorescent staining of the cells exploited a primary rabbit anti-ATF4 (D4B8) antibody followed by a solution containing a secondary Alexa Fluor 488-labeled goat anti-rabbit IgG along with Hoechst 33342 nuclei stain. In this way, confocal fluorescence microscopy at excitation wavelengths of 405 and 488 nm produced, as a direct primary readout, the intensity of ATF4 located in the nucleus as well as the ratio of ATF4 located between the nucleus (colocalized with Hoechst) and cytoplasm (see Figure 3B,C for representative images and the Supporting Information for channel-by-channel images). Concurrent readout of the number of stained nuclei (Hoechst nuclei stain) in live cells was also used as an internal cytotoxicity measure. This high-content method allowed detailed quantitative cellular dose responses to be determined with precision (Figure 3D). This also revealed that only the most potent DPAGT1 inhibitor, tunicamycin, caused a significant increase in ATF4 in the nucleus as a marker (EC_50_ = 44 ± 3.2 nM). Consistent with ATF4 as a driver of apoptosis, this also drove cell death at higher concentrations (EC_50_ ∼ 10 μM) similar to those determined previously.^33^ This therefore identified a clear dose–response window of more than 2 orders of magnitude over such key downstream events.

This combined use of an *in vitro* assay against DPAGT1 coupled with a high-content quantitative cellular assay allowed us to separate the critical flux of protein glycosylation from the induction of ER stress and consequent cell death. Analogues bearing the large lipid at the gating site (e.g., **Tun-9**_**E**_**,9**_**G**_ and **Tun-4**_**E**_**,9**_**G**_), which showed minimal DPAGT1 inhibition, did not induce any ER stress response in the phenotypic assay. Tunicamycin itself as a potent inhibitor of DPAGT1 exhibits a clear ER stress response as well as causing cell death. Graduated inhibitors **Tun-8**_**E**_**,2**_**G**_ and **Tun-9**_**E**_**,i5**_**G**_, which retain some DPAGT1 activity (35% and 55% of WT activity, respectively), did not induce ER stress. This suggests that even a background, low-level flux of protein N-glycosylation is sufficient to protect HEK cells from the UPR and avoid the onset of the apoptotic ER stress response. In turn, there must also be a so-called “biting point” at which DPAGT1 inhibition is sufficient to cause ER stress and subsequent cell death. Indeed, the clear dose–response curve for tunicamycin itself (Figure 3D), with a clear critical concentration at which the onset of ER stress begins, also supports this hypothesis. As noted above, this biting point is furthermore distinct from those that drive cell death. In this way, windows of dose-related cellular modulation may be considered. To probe the generality of this phenomenon, we also tested a targeted panel of modulated tunicamycin analogues (**Tun**, **Tun-8**_**E**_**,2**_**G**_, **Tun-8**_**E**_**,8**_**G**_, and **Tun-9**_**E**_**,9**_**G**_, representing strong, moderate, and minimal DPAGT1 inhibitors) in primary cells, namely, human dermal fibroblasts. We again saw a tuned response in which only **Tun** itself leads to an increase in ATF4 (Figures S8–S12).

Next, to relate *in vitro* DPAGT1 inhibition to cellular N-glycosylation, we used the same targeted panel of analogues (**Tun**, **Tun-8**_**E**_**,2**_**G**_, **Tun-8**_**E**_**,8**_**G**_, and **Tun-9**_**E**_**,9**_**G**_) to examine the specific glycosylation of a model glycoprotein (His-tagged IgG-Fc domain) when expressed in the presence of gradients of these tunicamycin analogues. Consistent with tuned potency toward DPAGT1 *in vitro*, we observed that both **Tun-8**_**E**_**,2**_**G**_ and **Tun** resulted in the expression of non-glycosylated IgG-Fc with clearly tuned inhibitory dose–response windows (**Tun** > **Tun-8**_**E**_**,2**_**G**_, as indicated by distinct reduction in molecular weight; Figure S13), while the other analogues resulted in expression of intact glycosylated protein. These observations valuably mechanistically coupled the effects of *in vitro* DPAGT1 inhibition tuning with *in cellulo* N-glycosylation tuning and hence the ER stress modulation demonstrated phenotypically.

Finally, we considered the possibility of other pathways downstream of our compound-mediated regulation of the key upstream enzyme DPAGT1 (and hence protein glycosylation). All three UPR pathways (Figure 1) are regulated by the intervening chaperone binding immunoglobulin protein (BiP);^34^ as a consequence, the triggers that we drive here through glycosylation deficits are likely to be essentially the same. Initial experiments suggest that IRE1 phosphorylation at Ser724 may be observed in HEK 293T cells (Figure S15), consistent with possible activation of this pathway also. It should be noted that the observation of ATF4 does not necessarily indicate commitment to an apoptotic outcome. CHOP is considered to be the key regulator of apoptosis on the dominant integrated stress response (ISR) pathway (PERK–ATF4–CHOP).^34^ To test this critical linkage of ATF4 to CHOP, we also determined the nuclear signal from CHOP in rat fibroblasts (NRK-49F) (Figure S14). These experiments revealed dose–response curves in which both ATF4 and CHOP were clearly correlated. Notably, while at 18 h essentially identical EC_50_ values were observed, at 6 h CHOP is still less established (as shown by a moderated potency), indicative of a gene product arising later than ATF4 and consistent with an ATF4-driven response. They also confirmed that stress response gene expression is relatively fast. Together these results supported our initial focus here on the dominant ISR pathway (PERK–ATF4–CHOP), yet we cannot discount the interesting possibility of associated pathways mediating the effects that we see here.

## Discussion

We have generated a panel of tuned DPAGT1 inhibitors based on the core scaffold of the natural product tunicamycin utilizing a semisynthetic approach beginning from the natural product at gram scale; our methods here focused on identified lipid modulation and complement those of base alteration aimed at targeting antibacterial functions.^35^ This panel was screened in an *in vitro* activity assay against DPAGT1, confirming the generation of a range of compounds that can induce graduated (from full to negligible) DPAGT1 activity and hence graduated *in cellulo* protein N-glycosylation. Our screening approach connected an “upstream” flux point (assessed by *in vitro* activity) to a “downstream” proxy (nuclear intensity of ATF4, a key transcriptional regulator of the ER stress response, which is itself translationally regulated^7^) using a high-content fluorescence phenotypic screen. This revealed that only more potent DPAGT1 inhibition induced ER stress; intermediate inhibitors of DPAGT1 did not.

In this way, we show a clearly defined relationship between inhibition of DPAGT1 (and thus shutdown of the N-glycosylation pathway^33^) and the onset of ER stress. Through systematic synthetic modulation of a privileged glycomimetic natural product scaffold, we have generated a “tuned” library of DPAGT1 inhibitors that modulate protein N-glycosylation activity at a central controlling flux point, yet without causing downstream ER stress. This suggests that even a background flux of N-glycosylation is sufficient to prevent the UPR from deviating toward an apoptotic ER stress pathway in healthy cells. Our methods also apparently delineate clear windows of dose–response between, e.g., ER stress and cell death.

Interestingly, recent studies have suggested that the parent natural product tunicamycin itself possesses selectivity toward certain cancerous cell types over nondiseased cells. In one instance, tunicamycin has been shown to aggravate ER stress in multidrug-resistant human gastric adenocarcinoma cells (SGC7901) beyond that in normal cells.^36^ Notably, these cells exhibit a basal level of ER stress above that of normal cells, which is thought to provide an initial level of cytoprotection. By selectively inducing apoptosis in cancerous cells that are already close to an apoptotic “tipping point” through exacerbating the basal level of ER stress already present, such small molecules may therefore provide an apparent additional lever for targeted cell killing. The quantitative determination of the windows between ER stress and cell death that we have demonstrated here in human cells, not only in robust cell lines (HEK293) but also in potentially relevant primary cells (human dermal fibroblasts), will aid precise knowledge of such “tipping points” and the potential creation of further compounds that will, for example, modulate this window.

Moreover, and more controversially, it has been suggested,^37^ perhaps counterintuitively, on the basis of compounds proposed to be more potent DPAGT1 inhibitors (assessed using unnatural DPAGT1 substrates) that native tunicamycins may not gain their cytotoxicity through DPAGT1 inhibition at all. Our results do not support this hypothesis insomuch as toxicity can be driven by ER stress—we see clear markers consistent with activation of the UPR response on a statistically validated sample scale. Nonetheless, we cannot discount the possibility that other modes of action may apply in differing phenotypes observed in varied cell types.

Therefore, together these and our observations highlight that the precise mode of cellular action that arises from manipulating the lynchpin glycan pathway enzyme DPAGT1 raises many open questions and necessitates the application of precise mechanistic tools that unpick subcellular pathways rather than relying on more broad characterization of phenotype (e.g., toxicity) alone—some of these tools have been suggested here. One particularly exciting avenue for future exploration will involve the use of the tuned analogues, for which we have shown proof of principle here and that exhibit low toxicity to *healthy* cells, in finding the more accessible “tipping point” in cancerous cells already under ER stress. Our initial applications here even in primary human cells suggest promise in this regard. Limited analogues explored to date instead appear to drive other phenotypes.^37^ This is the subject of ongoing studies using the methods that we have set out here.

## Supporting Information Available

Supplementary synthetic chemistry methods, supplementary biological methods, supplementary tables, and supplementary figures (PDF)
