# Supplementary material for: Lipid-Modulated, Graduated Inhibition of N-Glycosylation Pathway Priming Suggests Wide Tolerance of ER Proteostasis to Stress
Source: ACS Cent Sci. 2024 Dec 26;11(1):107–15. doi: 10.1021/acscentsci.4c01506 (PMC11758266; doi:10.1021/acscentsci.4c01506)
Supplement: Supplementary file 1 — oc4c01506_si_001.pdf [file oc4c01506_si_001.pdf]

Supporting Information

Lipid-Modulated, Graduated Inhibition of N-Glycosylation Pathway Priming Suggests Wide Tolerance of ER Proteostasis to Stress

Andrew M. Giltrap, Niamh Morris, Yin Dong, Stephen A. Cochrane, Thomas Krulle, Steven Hoekman, Martin Semmelroth, Carina Wollnik, Timea Palmai-Pallag, Elisabeth P. Carpenter, Jonathan Hollick, Alastair Parkes, York Rudhard and Benjamin G. Davis

SUPPLEMENTARY SYNTHETIC CHEMISTRY METHODS 4

General Experimental 4

General Methods 4

Synthesis of Tun Analogues 5

SUPPLEMENTARY BIOLOGICAL METHODS 18

DPAGT1 Expression Protocol 18

DPAGT1 Activity Assay 19

In cell glycoprotein inhibition assays 20

Immunoblotting 21

SUPPLEMENTARY PHENOTYPIC SCREENING METHODS 24

Method for HEK293. 24

Method for Primary Human Dermal Fibroblasts. 24

Method for ATF4-CHOP Correlation Evaluation in Rat Fibroblasts. 25

SUPPLEMENTARY TABLES 26

Table S1. % Activity of DPAGT1 in the presence of a tunicamycin analogue compared to a negative control (gel filtration buffer - GFB). 26

SUPPLEMENTARY FIGURES 27

Figure S1. Schematic Representation of the DPAGT1 radiometric assay. 27

Figure S2. Extracted data from the high-throughput immunofluorescence assay in HEK293 cells depicting nuclear intensity of ATF4 on the left y-axis, and the total live cell count on the right y-axis. 27

Figure S3. Extracted data from the high throughput immunofluorescence assay in HEK cells depicting nuclear:cytoplasmic ratio of ATF4 on the left y-axis, and the live cell count on the right y-axis. 28

Figure S4. Two channels used in the high-content immunofluorescence assay for tunicamycin in HEK cells. 28

Figure S5. Two channels used in the high-content immunofluorescence assay for Tun-9_E,9_G in HEK cells. 28

Figure S6. Two channels used in the high-content immunofluorescence assay for Tun-8_E,2_G in HEK cells. 29

Figure S7. Two channels used in the high-content immunofluorescence for DMSO assay in HEK cells. 29

Figure S8. Extracted data from the high-throughput immunofluorescence assay in human dermal fibroblasts depicting absolute nuclear intensity of ATF4 on the left y-axis, and the live cell count per field (14/well) on the right y-axis. 30

Figure S9. Extracted data from the high-throughput immunofluorescence assay in human dermal fibroblasts depicting nuclear:cytoplasmic ratio of ATF4 on the left y-axis, and the live cell count per field (14/well) on the right y-axis. **30**

Figure S10. Two channels used in the high-content immunofluorescence assay for tunicamycin in human dermal fibroblasts. **30**

Figure S11. Two channels used in the high-content immunofluorescence assay for Tun-9_E,9_G in human dermal fibroblasts. **31**

Figure S12. Two channels used in the high-content immunofluorescence assay for Tun-8_E,2_G in human dermal fibroblasts. **31**

Figure S13. SDS-Page and Western Blot analysis of a protein glycosylation assay in the presence of four lipid-altered tunicamycin variants. **33**

Figure S14. Correlation of CHOP with ATF4 Signal in Rat Fibroblasts. **34**

Figure S15. Western Blot analysis of IRE1 phosphorylation response to lipid-altered Tun-8_E,2_G variant. **35**

SUPPLEMENTARY REFERENCES 36

Supplementary Synthetic Chemistry Methods

General Experimental

Proton nuclear magnetic resonance (δ_H) spectra were recorded on a Bruker DPX 200 (200 MHz), Bruker DPX 400 (400 MHz), Bruker DQX 400 (400 MHz), Bruker AVC 500 (500 MHz) or Bruker AV 700 (700 MHz) spectrometer. Carbon nuclear magnetic resonance spectra were recorded on a Bruker DQX 400 (100 MHz) or Bruker AVC 500 (125 MHz) with a ^{13}C cryobprobe (125 MHz) AV 600 (151 MHz) with a ^{13}C cryobprobe (151 MHz) or AV 700 (176 MHz) with a ^{13}C cryobprobe (176 MHz). Spectra were assigned using a combination of ^1H, ^{13}C, HSQC, HMBC, COSY, and TOCSY. All chemical shifts were quoted on δ-scale in ppm, with residual solvent as internal standard. Coupling constants (*J*) are reported in hertz (Hz). Low-resolution mass spectra were recorded on a LCT Premier XE using electrospray ionization (ES). Thin layer chromatography (TLC) was performed on Merck EMD Kieselgel $60F_{254}$ precoated aluminum backed plates. TLC and RF-TLC were visualized in combination of: 254/365 nm UV lamp; sulfuric acid (2 M in EtOH/Water 1:1); ninhydrin (2% ninhydrin in EtOH); aqueous $KMnO_4$ (5% $KMnO_4$ in 1 M NaOH); aqueous phosphomolybdinc acid/Ce(IV) (2.5% phosphomolybdic acid hydrate, 1% cerium(IV) sulfate hydrate, and 6% H_2SO_4); or ammonium molybdate (5% in 2M H_2SO_4). Flash chromatography was carried out with Fluka Kiegselgel 60 220-440 mesh silica gel. All solvents (analytical or HPLC) used were purchased from Sigma Aldrich, Fisher Scientific, or Rathburn. Anhydrous solvents were purchased from Sigma Aldrich and stored over molecular sieves (<0.005 % H_2O). Petrol refers to the fraction of petroleum ether boiling point in the range of 40 – 60 °C. Brine refers to saturated solution of NaCl.

General Methods

HPLC Method A

Gradient: 30% → 95% B over 10 mins. 40 $mLmin^{-1}$

A = 0.1% Formic Acid in H_2O, B = 0.1% Formic Acid in MeCN

Column: Waters Sunfire C18, 30 x 100 mm, 10 μm

HPLC Method B

Gradient: 5% B → 65% B over 2 mins then 65% B for 28 mins. 20 $mLmin^{-1}$

A = 0.2% Ammonium Hydroxide in H_2O, B = MeCN

Column: Waters XBridge, 30 x 100 mm, 5 µm

HPLC Method C

Gradient: 5% B → 30% B over 0.5 mins then 30 → 40% B for 22 mins. 40 $mLmin^{-1}$

A = 0.2% Ammonium Hydroxide in H_2O, B = MeCN

Column: Waters XBridge, 30 x 100 mm, 5 µm

Synthesis of Tun Analogues

Tun(OAc)$_8$

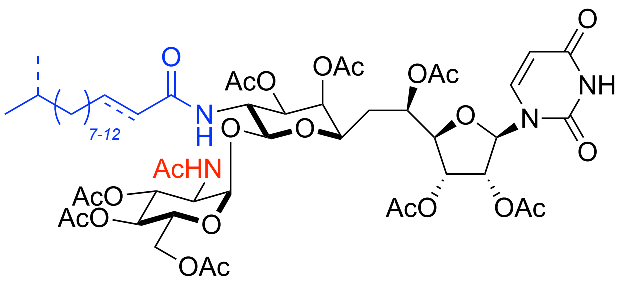

Tunicamycin (0.95 g, 1.1 mmol) was taken up in pyridine (5.0 mL) and acetic anhydride (3.0 mL, 32 mmol) was added. The reaction mixture was stirred overnight at rt and concentrated under reduced pressure. The material was obtained as a pale yellow solid in quantitative yield. The material was used without further purification.

LRMS (ESI) m/z calcd for $C_{55}H_{81}N_4O_{24}$ (C_{16} lipid chain) $[M+H]^+$: 1181.5, found: 1181.5.

Tun(OAc)$_8$(NBoc)$_3$

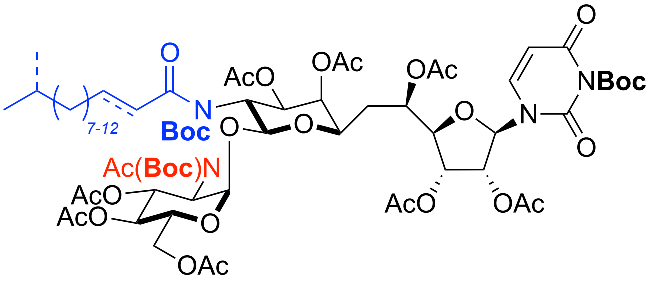

Tun(OAc)$_8$ (699 mg, 0.592 mmol) was dissolved in dry THF (10 mL) and DMAP (72.3 mg, 0.592 mmol) was added under a nitrogen atmosphere. The mixture was heated to 60 °C and 4 portions of di-tert-butyl dicarbonate (4 × 139 mg, 4 × 146 µL, 17.8 mmol) were added over the course of 2 h. After addition of the last portion, the reaction mixture was stirred for another 1 h and concentrated under reduced pressure. The crude was then resubmitted to the same reaction condition but without the addition of another portion of DMAP. The resulting solid was purified by flash column chromatography (1% MeOH in DCM) to yield the product (633 mg, 72% yield) as an orange solid.

The analysis is in agreement with the literature.

TLC: R$_f$ 0.5 in ethyl acetate/petrol (EtOAc/Petrol, 6:4); **^{1}H NMR** (500 MHz, $CDCl_3$) δ ppm 7.48 (d, $J_{6,5}$ = 8.0 Hz, 1 H, H-6^{uracil}), 6.89 (dt, $J_{HC=CH\ trans}$ = 15.1 Hz, J = 6.9 Hz, 1 H, C=C*H*-CH$_2$), 6.82 (dt, $J_{HC=CH\ trans}$ = 14.5 Hz, J = 7.6 Hz, 1 H, C=C*H*-CH2), 6.39 (d, $J_{HC=CH\ trans}$ = 15.1 Hz, 1 H, C=C*H*CO), 6.27 (d, $J_{HC=CH\ trans}$ = 15.4 Hz, 1 H, C=C*H*-CO), 6.11 (m, 1 H, C-H^{anomeric}), 5.85 (m, 1 H, CH^{anomeric}, H-5^{uracil}), 5.83 (d, J = 8.2 Hz, 1 H, H-5^{uracil}), 5.61 (dd, J = 11.5 Hz, J = 3.3 Hz, 1 H), 5.53 (dd, J = 11.3 Hz, J = 3.5 Hz, 1 H), 5.49 (d, J = 8.2 Hz, 1 H), 5.43 (m, 1 H), 5.29 – 5.35 (m, 1 H), 5.09 – 5.25 (m, 4 H), 5.01 – 4.10 (m, 1 H), 4.99 (d, J = 9.1 Hz), 4.94 (dd, J = 11.5 Hz, J = 3.3 Hz, 1 H), 4.91 (s, 1 H), 4.52 – 4.60 (m, 1 H), 4.30 – 4.39 (m, 1 H), 4.17 (d, J = 10.4 Hz, J = 2.2 Hz, 1 H), 4.05 – 4.10 (m, 1 H), 3.76 (dd, J = 8.8 Hz, J = 3.5 Hz, 1 H), 3.68 (dd, J = 9.9 Hz, J = 2.0 Hz, 1 H), 2.34 (s, 1 H), 2.29 (s, 2 H, C*H*$_3$^{NHAc}), 2.27 (s, 1 H, C*H*$_3$^{NHAc}), 1.87 – 2.22 (m, 24H, 8 x CH$_3$^{Ac}), 1.59, 1.56, 1.55, 1.53, 1.52 (5 x s, 27 H, 9 x CH$_3$^{Boc}), 1.40 (m, 13 H), 1.08 – 1.18 (m, 2 H, CH$_2$^{acyl}), 0.86, 0.85 (2 x s, 2 x 3 H, C*H*$_3$^{acyl}); **^{13}C NMR** (126 MHz, CD_3OD) δ ppm 177.4, 177.5, 170.8,170.7, 170.1, 169.9, 169.7, 169.6, 169.5, 169.4, 169.1 (C=O), 168.2 (C-4 C=O), 159.8 (C-2 C=O), 153.0, 152.9, 157.7, 152.7, 152.1, 148.3, 148.1, 147.3, 139.1, 139.0, 138.9 (C=*C*H-CH$_2$, C-6^{uracil}), 124.4, 123.9 (C=*C*H-CO), 103.5, 103.2 (C-1’’), 97.8, 87.7, 86.9, 87.0, 86.9, 86.3, 82.4, 82.3, 72.1, 70.4, 70.2, 70.1, 69.6, 69.5, 69.4, 69.2, 69.1, 68.8, 68.0, 67.9, 61.5, 61.4, 57.657.0, 54.8, 39.0, 38.5, 36.6, 34.3, 32.7, 32.5,

32.4, 31.9, 29.9, 29.6, 29.5, 29.4, 29.3, 29.2, 28.2, 28.0, 27.9, 27.8, 27.6, 27.4, 22.6, 20.9, 20.9, 20.7, 20.6, 20.5, 20.4 (C-1'), 84.1 (C-4'), 73.5 (C-2'), 72.2 (C-3"), 72.2 (C-9'), 71.7 (C-7'), 70.9 (C-3'), 70.8, 70.3 (C-5', C-8'), 69.8 (C-5"), 69.7 (C-4"), 63.0 (C-6"), 52.6 (C-2"), 51.8 (C-10'), 40.3 (-CH$_2$CH(CH$_3$)$_2$), 33.2 (-CH$_2$CH=C), 33.1 (C-6'), 30.3 - 31.1 (5 x C, 5 x -CH_2^{acyl}), 29.4 (-CH$_2$CH$_2$CH=C), 29.2 (-CH(CH$_3$)$_2$), 28.6 (-CH_2^{acyl}), 23.0, 23.1 (2 x C, -CH(CH$_3$)$_2$), 22.9 (-CH_3^{NHAc}), 21.1 (-CH_3^{Ac}), 20.7 (2 x C, 2 x -CH_3^{Ac}), 20.6, 20.6, 20.6, 20.6, 20.3 (5 x C, 5 x -CH_3^{Ac}) **IR** ν: 2928, 2361, 2341, 1743, 1686, 1369, 1218, 1143, 1029; **LRMS** *m/z* (ESI$^+$): 1503 [(M+Na)$^+$, 100%]; (ESI$^-$): 1515 [(M+Cl)$^-$, 100%]. Flanking peaks with mass ± 14 corresponded to 8 x CH$_2$, 9 x CH$_2$, 10 x CH$_2$, and 11 x CH$_2$.

Tun(NBoc)$_3$

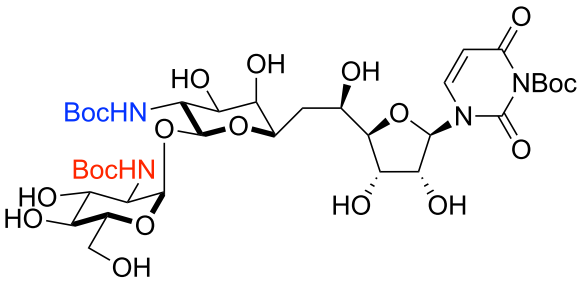

Tun(OAc)$_8$(NBoc)$_3$ (165 mg, 0.112 mmol) was dissolved in MeOH (500 µL) and freshly prepared NaOMe (0.5 M solution in MeOH, 112 µL, 0.0562 mmol) was added. The reaction was stirred for 2 h at rt and initially followed by TLC (EtOAc/heptane, 4:1) and later by LCMS (MSQ2). The deacetylation was noticeably faster than the deamidation. Upon completion, water (100 µL) was added the reaction mixture was neutralised with amberlite IR120 acidic resin and filtered off. The filtrate was concentrated under reduced pressure. The material was taken through without further purification, because the Boc group on the uracil moiety is unstable on the column (HPLC also).

LRMS (ESI) m/z calcd for $C_{36}H_{59}N_4O_{20}$ [M+H]$^+$: 867.4, found: 867.7.

Tun-0$_E$,0$_G$

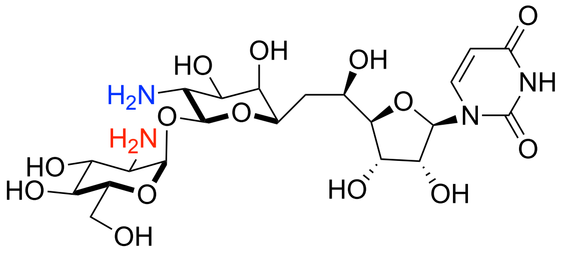

Tun(NBoc)$_3$ (crude from previous step) was taken up in MeOH (5.0 mL), acidified with 4 M HCl dioxane solution (5.0 mL) and stirred for 1 h at rt. The mixture was concentrated taken up in 0.5 M aqueous HCl (1 mL) and concentrated again. The crude was purified by reverse phase flash column chromatography (95:5, water (0.5% formic acid)/acetonitrile (0.5% formic acid)) to yield the title compound as a pale yellow solid (49 mg, 68% yield over two steps).

^{1}H NMR (700 MHz, D_2O) δ ppm 7.82 (d, *J* = 8.2 Hz, 1 H, H-6), 5.87 (d, *J* = 8.2 Hz, 1 H, H-5) 5.86 (d, *J* = 5.3 Hz, 1 H, H-1'), 5.53 (d, *J* = 3.4 Hz, 1 H, H-1''), 5.00 (d, *J* = 8.3 Hz, 1 H, H-11'), 4.31 - 4.26 (m, 2 H, H-2', H-3'), 4.06 (td, *J* = 2.6, 11.1 Hz, 1 H, H-5'), 3.94 (dd, *J* = 3.3, 11.0 Hz, 1 H, H-9'), 3.92 - 3.87 (m, 3 H, H-7', H-3'', H-5''), 3.84 (d, *J* = 3.2 Hz, 1 H, H-8'), 3.79 (dd, *J* = 3.8, 12.5 Hz, 1 H, H-6''), 3.70 (dd, *J* = 2.2, 12.4 Hz, 1 H, H6''), 3.57 (t, *J* = 9.6 Hz, 1 H, H-4''), 3.39 (dd, *J* = 3.5, 10.8 Hz, 1 H, H-2''), 3.31 (dd, *J* = 8.4, 11.0 Hz, 1 H, H-10'), 1.97 (ddd, *J* = 2.0, 10.4, 14.6 Hz, 1 H, H-6'), 1.70 - 1.64 (dtd, *J* = 2.8, 11.2 Hz, 1 H, H-6'); **^{13}C NMR** (176 MHz, CD_3OD) δ ppm 166.17 (C-4), 151.8 (C-2), 142.0 (C-6), 102.4 (C-5), 99.4 (C-11'), 97.0 (C-1''), 88.7 (C-1'), 86.9 (C-4'), 73.4 (C-2'), 73.2 (C-5''), 71.8 (C-7''), 69.5 (C-8'), 69.3 (C-3''), 69.2 (C-9'), 68.9 (C-4'), 68.7 (C-3'), 66.9 (C-5'), 59.8 (C-6''), 53.8 (C-2''), 53.0 (C-10'), 33.3 (C-6'); **IR** (neat) ν: 3295 (N-H, O-H), 3057 (=C-H), 2922 (-C-H), 1673 (C=O), 1263 (C-N), 1109 (C-O), 1064 (C-O); **LRMS** m/z (ESI$^+$): 567 [(M+H)$^+$, 100%]; **HRMS** m/z (ESI$^+$): calc. $C_{21}H_{35}N_4O_{14}$ (M+H)$^+$ = 567.2144, found 567.2136.

Tun-8E,8G

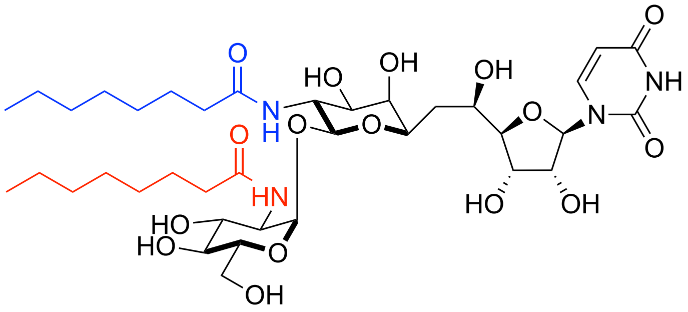

A solution of HATU (61 mg, 0.1609 mmol), octanoic acid (24 µL, 0.1658 mmol) and DIPEA (80 µL, 0.4598 mmol) in DMF (300 µL) was prepared and transferred to a vial containing **Tun-0E,0G** (49 mg, 0.0766 mmol) and DMF (350 µL). The reaction mixture was stirred for 2 h at rt. Then the mixture was concentrated under reduced pressure and purified by HPLC (Method A) to yield **Tun-8E,8G** as a white solid (24 mg, 38% yield).

LRMS (ESI) m/z calcd for $C_{37}H_{63}N_4O_{16}$ $[M+H]^+$: 819.4, found: 819.6. **^{1}H NMR** (500 MHz, Methanol-d4): δ 7.81 (d, *J* = 8.1 Hz, 1H), 5.82 (d, *J* = 5.9 Hz, 1H), 5.65 (d, *J* = 8.1 Hz, 1H), 4.84 (d, *J* = 3.5 Hz, 1H), 4.50 (d, *J* = 8.5 Hz, 1H), 4.15 – 4.04 (m, 2H), 3.98 – 3.88 (m, 2H), 3.85 (dd, *J* = 10.1, 8.6 Hz, 1H), 3.80 (dd, *J* = 10.6, 3.5 Hz, 1H), 3.77 – 3.70 (m, 2H), 3.66 (dd, *J* = 10.8, 1.9 Hz, 1H), 3.61 – 3.49 (m, 4H), 2.29 – 2.03 (m, 4H), 2.00 (ddd, *J* = 12.9, 10.8, 2.0 Hz, 1H), 1.60 – 1.47 (m, 4H), 1.44 (ddd, *J* = 14.0, 11.4, 2.5 Hz, 1H), 1.32 – 1.15 (m, 16H), 0.85 – 0.76 (m, 6H). This data is in agreement with Dong et. al.[1]

Tun-9E,9G

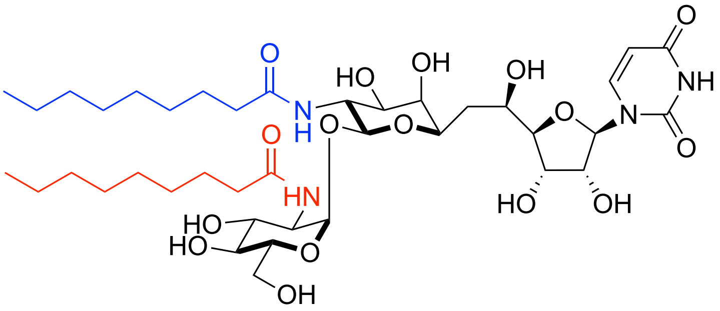

A solution of HATU (125 mg, 0.328 mmol), nonanoic acid (54.6 µL, 0.313 mmol) and DIPEA (164 µL, 0.938 mmol) in DMF (500 µL) was prepared and transferred to a vial containing **Tun-0E,0G** (100 mg, 0.156 mmol) and DMF (800 µL). The reaction mixture was stirred for 2 h at rt. Then the mixture was concentrated and purified by HPLC (Method A). The resulting beige

solid was carefully washed with CH_2Cl_2 to **Tun-9$_E$,9$_G$** as an off-white solid (17.5 mg, 13% yield).

LRMS (ESI) m/z calcd for $C_{39}H_{67}N_4O_{16}$ $[M+H]^+$: 847.5, found: 847.7. **^{1}H NMR** (500 MHz, Methanol-d4): δ 7.91 (d, *J* = 8.1 Hz, 1H), 5.92 (d, *J* = 5.9 Hz, 1H), 5.75 (d, *J* = 8.1 Hz, 1H), 4.94 (d, *J* = 3.4 Hz, 1H), 4.60 (d, *J* = 8.5 Hz, 1H), 4.25 – 4.15 (m, 2H), 4.09 – 3.98 (m, 2H), 3.98 – 3.92 (m, 1H), 3.90 (dd, *J* = 10.6, 3.5 Hz, 1H), 3.87 – 3.79 (m, 2H), 3.76 (dd, *J* = 10.8, 2.0 Hz, 1H), 3.72 – 3.59 (m, 4H), 2.33 (ddd, *J* = 14.6, 9.6, 6.4 Hz, 1H), 2.28 – 2.15 (m, 4H), 2.10 (ddd, *J* = 12.8, 10.7, 1.9 Hz, 1H), 1.71 – 1.57 (m, 4H), 1.53 (ddd, *J* = 13.9, 11.3, 2.5 Hz, 1H), 1.32 (s, 20H), 0.96 – 0.84 (m, 6H). This data is in agreement with Dong et. al.[1]

Tun(OAc)$_8$(NBoc)$_2$

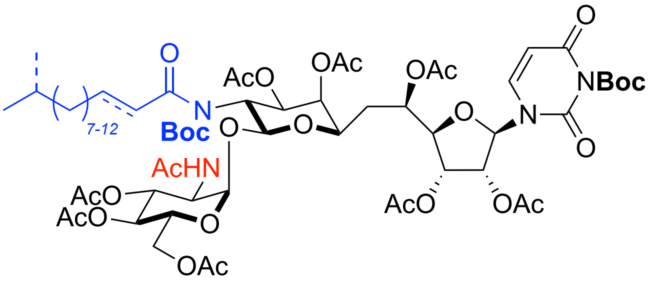

Tun(OAc)$_8$ (650 mg, 0.507 mmol) was dissolved in dry THF (8 mL) and DMAP (62 mg, 0.507 mmol) and di-tert-butyl dicarbonate (221 mg, 1.01 mmol) were added and the resulting mixture was heated to 60 °C. After 1 h the progress was checked by TLC and the reaction had not gone to completion, therefore an additional portion of di-tert-butyl dicarbonate (170 mg, 0.779 mmol) was added and the reaction was stirred for another 1 h. Subsequently, the mixture was quenched with water and concentrated under reduced pressure. Flash column chromatography (CH_2Cl_2/MeOH, 1:0 to 9:1) yielded the Boc-protected compound (**Tun(OAc)$_8$(NBoc)$_2$**, mixture of mostly two regioisomers) as an orange solid (618 mg, 88% yield). R_f = 0.51 in EtOAc/heptane 4:1.

Tun-0$_E$,2$_G$(NBoc)$_2$

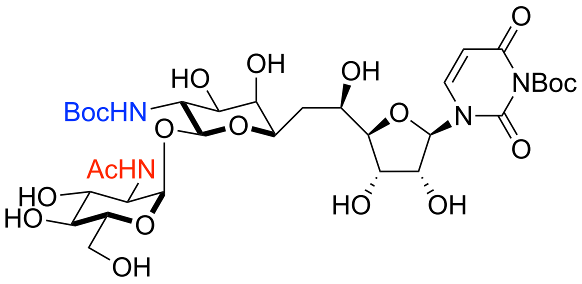

Tun(OAc)$_8$(NBoc)$_2$ (mixture of mostly two regioisomers, 618 mg, 0.447 mmol) was dissolved in dry MeOH (10 mL) and freshly prepared NaOMe (0.5 M sol in MeOH, 447 µL, 0.224 mmol) was added. The reaction mixture was stirred for 2 h at rt and initially followed by TLC (EtOAc/heptane, 4:1) and later by LCMS. Upon completion, water (100 µL) was added and the mixture was neutralised with amberlite IR120 acidic resin, filtered off and concentrated under reduced pressure to furnish the crude product (465 mg, 85% yield) as an orange solid. The material was taken through without further purification, because the Boc group on the uracil moiety is unstable on the column (HPLC also).

LRMS (ESI) m/z calcd for $C_{33}H_{53}N_4O_{19}$ $[M+H]^+$: 809.3, found: 809.4.

Tun-0$_E$,2$_G$

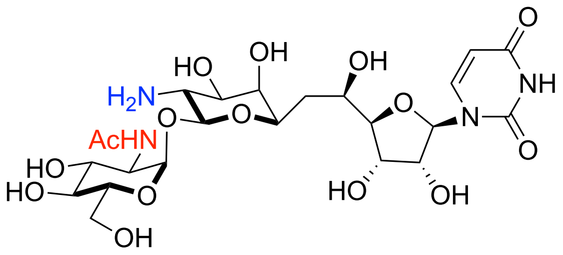

Tun-0$_E$,2$_G$(NBoc)$_2$ (crude from previous step, 25% scale) was taken up in MeOH (5.0 mL), acidified with 4 M HCl dioxane solution (5.0 mL) and stirred for 1 h at rt. The mixture was concentrated taken up in 0.5 M aqueous HCl (1 mL) and concentrated again. The crude was purified by reverse phase flash column chromatography (95:5, water (0.5% formic acid)/acetonitrile (0.5% formic acid)) to yield the title compound as a pale yellow solid (27 mg, 34% yield over two steps).

LRMS (ESI) m/z calcd for $C_{23}H_{37}N_4O_{15}$ $[M+H]^+$: 609.2, found: 609.5.

Tun-8$_E$,2$_G$

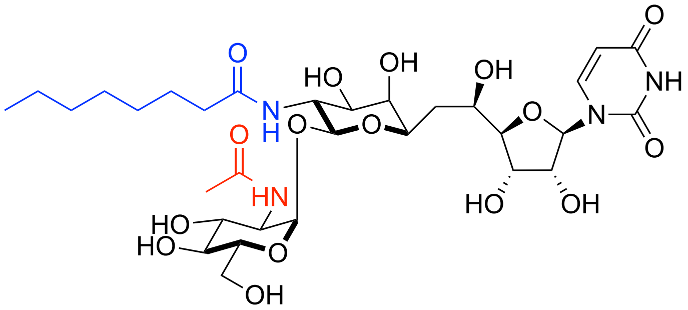

A solution of HATU (109 mg, 0.286 mmol), octanoic acid (45.3 μL, 0.286 mmol) and DIPEA (150 μL, 0.858 mmol) in DMF (600 μL) was prepared and transferred to a vial containing **Tun-0$_E$,2$_G$** (123 mg, 0.191 mmol) and DMF (400 μL). The reaction mixture was stirred for 2 h at rt. When the reaction was completed, the mixture was concentrated under reduced pressure, purified by reverse phase flash column chromatography (9:1 to 0:1, water (0.5% formic acid)/acetonitrile (0.5% formic acid)) and purified again by prep HPLC (SAS, acidic) to yield **Tun-8$_E$,2$_G$** as a white solid (15.5 mg, 11% yield).

LRMS (ESI) m/z calcd for $C_{31}H_{54}N_4O_{16}$ $[M+H]^+$: 735.3, found: 735.4. **^{1}H NMR** (500 MHz, Methanol-d_4) δ 7.90 (d, J = 8.1 Hz, 1H, H-6), 5.92 (d, J = 5.9 Hz, 1H, H-1'), 5.75 (d, J = 8.1 Hz, 1H, H-5), 4.94 (d, J = 3.5 Hz, 1H, H-1''), 4.61 (d, J = 8.5 Hz, 1H, H-11'), 4.22 (dd, J = 5.5, 3.0 Hz, 1H, H-3'), 4.19 (dd, 1H, H-2'), 4.06 – 3.98 (m, 2H, H-5', H-5''), 3.95 (dd, J = 9.9, 8.6 Hz, 1H, H-10'), 3.88 (dd, J = 10.6, 3.5 Hz, 1H, H-2''), 3.86 – 3.81 (m, 2H, H-4', H-6''), 3.76 (dd, J = 10.7, 2.0 Hz, 1H, H-7'), 3.71 – 3.58 (m, 4H, H-8',H-9', H-3'', H-6''), 3.35 – 3.33 (m, 1H, H-4''), 2.27 – 2.15 (m, 2H,$CH_2^{octanoyl}$), 2.10 (ddd, 1H, H-6'), 2.02 (s, 3H, Ac), 1.70 – 1.58 (m, 2H, 2 x $CH_2^{octanoyl}$), 1.53 (ddd, J = 13.9, 11.2, 2.3 Hz, 1H, H-6'), 1.42 – 1.21 (m, 8H, 4 x $CH_2^{octanoyl}$), 0.95 – 0.82 (m, 3H, $CH_3^{octanoyl}$).

11 protons exchanged, H-4'' obstructed by methanol signal

Tun-9$_E$,4$_G$(OAc)$_8$ and Tun-4$_E$,9$_G$(OAc)$_8$

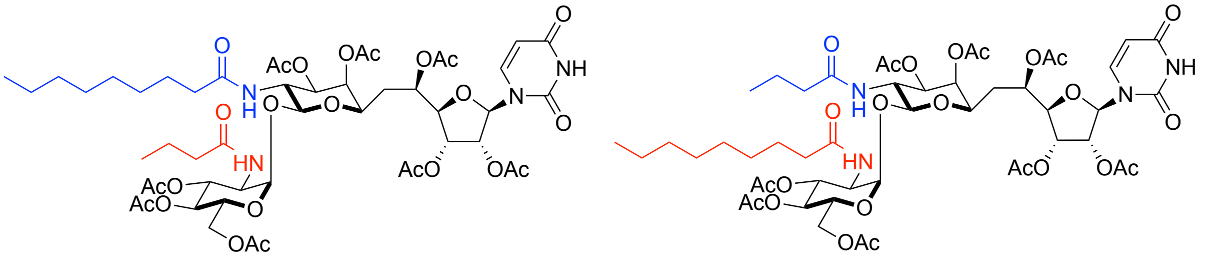

HATU (140 mg, 0.368 mmol), nonanoic acid (29 µL, 0.167 mmol), butanoic acid (15 µL, 0.167 mmol) and DIPEA (175µL, 1.004 mmol) were added to a solution of **Tun-0$_E$,0$_G$** (107 mg, 0.167) in DMF (1.50 mL). The reaction mixture was stirred for 1 h at rt. Upon completion, the mixture was concentrated under reduced pressure. The crude was purified by reverse phase flash column chromatography (9:1 to 0:1, water+formic acid/CH_3CN+formic acid) to give the intermediate 8-OH as a pale yellow solid (mixture of regioisomers, 22 mg, 17% yield).

LRMS (ESI) m/z calcd for $C_{34}H_{57}N_4O_{16}$ $[M+H]^+$: 777.4, found: 777.5.

The mixture (22 mg, 0.0283 mmol) was taken up in pyridine (400 µL) and acetic anhydride (240 µL) was added. The reaction mixture was stirred at rt for 5 h and concentrated under reduced pressure to yield the crude as a yellow solid (32 mg). The material was purified by HPLC (method B) to give regioisomer 1 (11.1 mg, 0.00997 mmol) and regioisomer 2 (10.6 mg, 0.00952 mmol) as white solids.

LRMS (ESI) m/z calcd for $C_{50}H_{74}N_4O_{24}$ $[M+2H]^{2+}$: 557.2, found: 557.3.

Tun-9$_E$,4$_G$

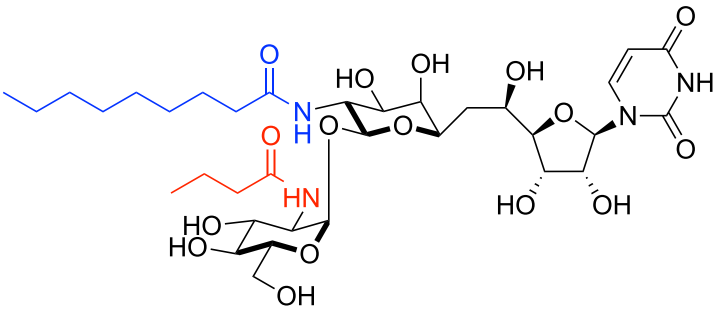

The acetylated starting material (regioisomer 1 (shorter retention time) of the regioisomer mixture, 11.1 mg, 0.00997 mmol) was dissolved in MeOH (1.00 mL) and freshly prepared NaOMe (0.5 M sol in MeOH, 19.9 µL, 0.00997 mmol) was added. The reaction mixture was

stirred at rt for 24 h. Upon completion of the OAc-deprotection, the reaction mixture was neutralised with amberlite IR120 resin, filtered, concentrated and washed with heptane to give **Tun-9$_E$,4$_G$** as a pale brown solid (4.9 mg, 63% yield).

LRMS (ESI) m/z calcd for $C_{34}H_{57}N_4O_{16}$ $[M+H]^+$: 777.4, found: 777.5. **^{1}H NMR** (500 MHz, MeOD) δ 7.90 (d, *J* = 8.1 Hz, 1H, H-6), 5.92 (d, *J* = 5.9 Hz, 1H, H-1'), 5.75 (d, *J* = 8.1 Hz, 1H, H-5), 4.94 (d, *J* = 3.3 Hz, 1H, H-1''), 4.61 (d, *J* = 8.5 Hz, 1H, H-11'), 4.22 (dd, *J* = 5.4, 2.9 Hz, 1H, H-3'), 4.20 – 4.16 (m, 1H, H-2'), 4.07 – 3.97 (m, 2H, H-5', H-5''), 3.95 (dd, *J* = 10.3, 8.6 Hz, 1H, H-10'), 3.91 (dd, *J* = 10.6, 3.4 Hz, 1H, H-2''), 3.88 – 3.80 (m, 2H, H-4', H-6''), 3.79 – 3.74 (m, 1H, H-7'), 3.72 – 3.61 (m, 4H, H-8',H-9', H-3'', H-6''), 3.46 – 3.32 (m, 1H, H-4''), 2.44 – 2.14 (m, 4H, $CH_2^{butanoyl}$, $CH_2^{nonanoyl}$), 2.14 – 2.02 (m, 1H, H-6'), 1.77 – 1.57 (m, 4H, $CH_2^{butanoyl}$, $CH_2^{nonanoyl}$), 1.57 – 1.45 (m, 1H, H-6'), 1.41 – 1.14 (m, 10H, 5 x $CH_2^{nonanoyl}$), 0.97 (t, *J* = 7.4 Hz, 3H, $CH_3^{butanoyl}$), 0.94 – 0.74 (m, 3H, $CH_3^{nonanoyl}$).

11 protons exchanged, H-4'' obstructed by methanol signal

Tun-4$_E$,9$_G$

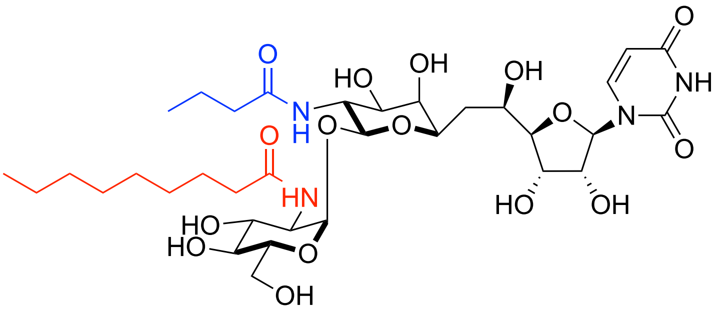

The acetylated starting material (regioisomer 2 (longer retention time) of the regioisomer mixture, 10.6 mg, 0.00952 mmol) was dissolved in MeOH (1.00 mL) and freshly prepared NaOMe (0.5 M sol in MeOH, 19.0 µL, 0.00952 mmol) was added. The reaction mixture was stirred at rt for 24 h. Upon completion of the OAc-deprotection, the reaction mixture was neutralised with amberlite IR120 resin, filtered, concentrated and washed with heptane to give **Tun-4$_E$,9$_G$** as a pale brown solid (4.9 mg, 66% yield).

LRMS (ESI) m/z calcd for $C_{34}H_{57}N_4O_{16}$ [M+H]+: 777.4, found: 777.5. **^{1}H NMR** (500 MHz, MeOD) δ 7.91 (d, J = 8.1 Hz, 1H, H-6), 5.92 (d, J = 5.9 Hz, 1H, H-1'), 5.75 (d, J = 8.1 Hz, 1H, H-5), 4.95 (d, J = 3.4 Hz, 1H, H-1''), 4.62 (d, J = 8.5 Hz, 1H, H-11'), 4.22 (dd, J = 5.4, 3.0 Hz, 1H, H-3'), 4.20 – 4.16 (m, 1H, H-2'), 4.07 – 3.98 (m, 2H, H-5', H-5''), 3.95 (dd, J = 10.5, 8.6 Hz, 1H, H-10'), 3.90 (dd, J = 10.5, 3.4 Hz, 1H, H-2''), 3.87 – 3.80 (m, 2H, H-4', H-6''), 3.80

– 3.73 (m, 1H, H-7'), 3.71 – 3.61 (m, 4H, H-8',H-9', H-3'', H-6''), 3.36 – 3.31 (m, 1H, H-4''), 2.33 (ddd, J = 15.9, 9.5, 6.5 Hz, 1H, 0.5 x $CH_2^{nonanoyl}$), 2.27 – 2.15 (m, 3H, 0.5 x $CH_2^{nonanoyl}$, $CH_2^{butanoyl}$), 2.14 – 2.00 (m, 1H, H-6'), 1.74 – 1.48 (m, 5H, H-6', $CH_2^{nonanoyl}$, $CH_2^{butanoyl}$), 1.43 – 1.13 (m, 10H, 5 x $CH_2^{nonanoyl}$), 0.96 (t, J = 7.4 Hz, 3H, $CH_3^{butanoyl}$), 0.93 – 0.79 (m, 3H, $CH_3^{nonanoyl}$).

11 protons exchanged, H-4'' obstructed by methanol signal

Tun-9$_E$,i5$_G$(OAc)$_8$ and Tun-i5$_E$,9$_G$(OAc)$_8$

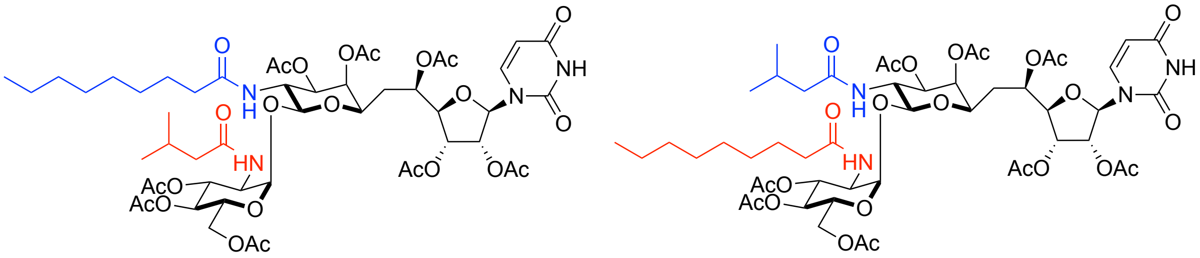

HATU (252 mg, 0.664 mmol), nonanoic acid (53 µL, 0.3018 mmol), isovaleric acid (33 µL, 0.3018 mmol) and DIPEA (316 µL, 1.811 mmol) were added to a solution of **Tun-0$_E$,0$_G$** (193 mg, 0.3018) in DMF (3 mL). The reaction mixture was stirred for 1 h at rt. Upon completion, the mixture was concentrated under reduced pressure. The crude was purified by reverse phase flash column chromatography (9:1 to 0:1, water+formic acid/CH3CN+formic acid) to give the intermediate 8-OH as a white solid (mixture of regioisomers, 70 mg, 29% yield).

LRMS (ESI) m/z calcd for $C_{35}H_{59}N_4O_{16}$ $[M+H]^+$: 791.4, found: 791.5.

The mixture (70 mg, 0.0885 mmol) was taken up in pyridine (1.00 mL) and acetic anhydride (600 µL) was added. The reaction mixture was stirred at rt for 5 h and concentrated under reduced pressure to yield the crude as a yellow solid (100 mg). The material was purified by HPLC (method C) to give regioisomer 1 (11.1 mg, 0.00997 mmol) and regioisomer 2 (10.6 mg, 0.00952 mmol) as white solids.

LRMS (ESI) m/z calcd for $C_{51}H_{76}N_4O_{24}$ $[M+2H]^{2+}$: 564.2, found: 564.3.

Tun-9$_E$,i5$_G$

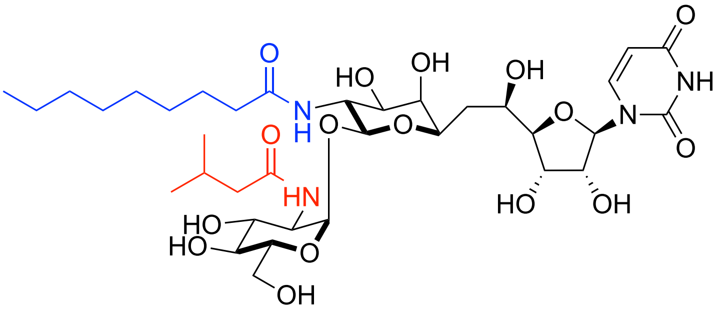

The acetylated starting material (regioisomer 1 (shorter retention time) of the regioisomer mixture, 11.7 mg, 0.0104 mmol) was dissolved in MeOH (350 µL) and freshly prepared NaOMe (0.5 M sol in MeOH, 11.4 µL, 0.0057 mmol) was added. The reaction mixture was stirred at rt for 3 d. Upon completion of the OAc-deprotection, the reaction mixture was neutralised with amberlite IR120 resin, filtered and concentrated to give **Tun-9$_E$,i5$_G$** as a white solid (7.4 mg, 90% yield).

LRMS (ESI) m/z calcd for $C_{35}H_{59}N_4O_{16}$ $[M+H]^+$: 791.4, found: 791.4. **^{1}H NMR** (500 MHz, MeOD) δ 7.90 (d, *J* = 8.1 Hz, 1H, H-6), 5.92 (d, *J* = 5.9 Hz, 1H, H-1'), 5.75 (d, *J* = 8.1 Hz, 1H, H-5), 4.95 (d, *J* = 3.5 Hz, 1H, H-1''), 4.61 (d, *J* = 8.5 Hz, 1H, H-11'), 4.22 (dd, *J* = 5.5, 2.9 Hz, 1H, H-3'), 4.18 (d, *J* = 5.6 Hz, 1H, H-2'), 4.08 – 3.97 (m, 2H, H-5', H-5''), 3.99 – 3.88 (m, 2H, H-10', H-2''), 3.88 – 3.80 (m, 2H, H-4', H-6''), 3.80 – 3.73 (m, 1H, H-7'), 3.72 – 3.60 (m, 4H, H-8',H-9', H-3'', H-6''), 3.43 – 3.32 (m, 1H, H-4''), 2.28 – 2.14 (m, 3H, $CH_2^{nonanoyl}$, 0.5 $CH_2^{isovaleroyl}$), 2.17 – 2.01 (m, 3H, H-6', $CH^{isovaleroyl}$, 0.5 $CH_2^{isovaleroly}$), 1.76 – 1.58 (m, 2H, 1 x $CH_2^{nonanoyl}$), 1.58 – 1.49 (m, 1H, H-6'), 1.40 – 1.16 (m, 10H, 5 x $CH_2^{nonanoyl}$), 1.05 – 0.94 (m, 6H, 2 x $CH_3^{isovaleroyl}$), 0.94 – 0.84 (m, 3H, $CH_3^{nonanoyl}$).

11 protons exchanged, H-4'' obstructed by methanol signal

Tun-i5$_E$,9$_G$

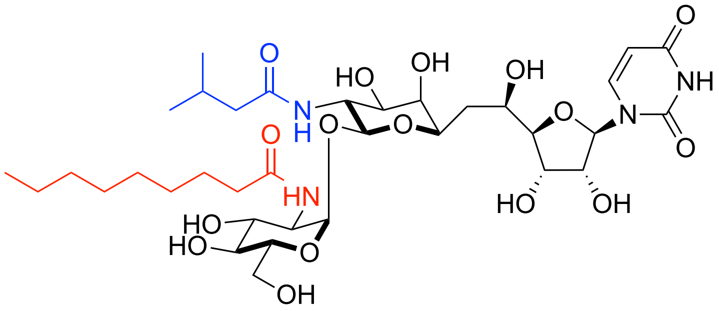

The acetylated starting material (purified regioisomer 2 (longer retention time) of the regioisomer mixture, 19.8 mg, 0.0176 mmol) was dissolved in MeOH (700 µL) and freshly prepared NaOMe (0.5 M sol in MeOH, 35.2 µL, 0.0176 mmol) was added. The reaction mixture was stirred at rt for 3 d. Upon completion of the OAc-deprotection, the reaction mixture was neutralised with amberlite IR120 resin, filtered and concentrated to give **Tun-i5$_E$,9$_G$** as a white solid (13.8 mg, 99% yield).

LRMS (ESI) m/z calcd for $C_{35}H_{59}N_4O_{16}$ $[M+H]^+$: 791.4, found: 791.4. **^{1}H NMR** (500 MHz, MeOD) δ 7.91 (d, *J* = 8.1 Hz, 1H, H-6), 5.92 (d, *J* = 5.9 Hz, 1H, H-1')), 5.75 (d, *J* = 8.1 Hz, 1H, H-5), 4.96 (d, *J* = 3.5 Hz, 1H, H-1''), 4.61 (d, *J* = 8.5 Hz, 1H, H-11'), 4.22 (dd, *J* = 5.4, 3.0 Hz, 1H, H-3'), 4.22 – 4.15 (m, 1H, H-2'), 4.07 – 3.97 (m, 2H, H-5', H-5''), 3.95 (dd, *J* = 10.5, 8.5 Hz, 1H, H-10'), 3.93 – 3.87 (m, 1H, H-2''), 3.88 – 3.80 (m, 2H, H-4', H-6''), 3.79 – 3.72 (m, 1H, H-7'), 3.72 – 3.59 (m, 4H, H-8',H-9', H-3'', H-6''), 3.42 – 3.32 (m, 1H, H-4''), 2.40 – 2.28 (m, 1H, 0.5 x $CH_2^{nonanoyl}$), 2.23 (ddd, *J* = 14.7, 9.4, 5.9 Hz, 1H, 0.5 x $CH_2^{nonanoyl}$), 2.16 – 2.00 (m, 4H, H-6', $CH^{isovaleroyl}$, $CH_2^{isovaleroyl}$), 1.82 – 1.49 (m, 3H, H-6', $CH_2^{nonanoyl}$), 1.45 – 1.14 (m, 10H, 5 x $CH_2^{nonanoyl}$), 0.97 (d, *J* = 6.2 Hz, 6H, 2 x $CH_3^{isovaleroyl}$), 0.94 – 0.86 (m, 3H, $CH_3^{nonanoyl}$).

11 protons exchanged, H-4'' obstructed by methanol signal

Supplementary Biological Methods

DPAGT1 Expression Protocol

Sequence

N-terminal 6-His, TEV cleavage site (underlined)

<u>MGHHHHHHSSGVDLGTENLYFQS</u>MWAFSELPMPLLINLIVSLLGFVATVTLIPAFRG
HFIAARLCGQDLNKTSRQQIPESQGVISGAVFLIILFCFIPFPFLNCFVKEQCKAFPHHE
FVALIGALLAICCMIFLGFADDVLNLRWRHKLLLPTAASLPLLMVYFTNFGNTTIVVP
KPFRPILGLHLDLGILYYVYMGLLAVFCTNAINILAGINGLEAGQSLVISASIIVFNLVE
LEGDCRDDHVFSLYFMIPFFFTTLGLLYHNWYPSRVFVGDTFCYFAGMTFAVVGILG
HFSKTMLLFFMPQVFNFLYSLPQLLHIIPCPRHRIPRLNIKTGKLEMSYSKFKTKSLSFL
GTFILKVAESLQLVTVHQSETEDGEFTECNNMTLINLLLKVLGPIHERNLTL
LLLLLQILGSAITFSIRYQLVRLFYDV

Cloning and expression

The WT DPAGT1 cDNA sequence was cloned into the pFB-LIC-Bse expression vector (available from the SGC) with an N-terminal purification tag with a tobacco etch virus (TEV) protease cleavage site, and a 6x His purification sequence. Baculoviruses were produced by transformation of DH10Bac cells. *Spodoptera frugiperda* (Sf9) insect cells in Sf-900 II SFM medium (Thermo Fisher) were infected with recombinant baculovirus and incubated for 65 h at 27 °C in shaker flasks.

Purification of DPAGT1 protein for structural and functional studies

Cell pellets from 1 litre of insect cell culture were resuspended in 40mL in lysis buffer (50 mM HEPES, pH 7.5, 5 mM $MgCl_2$, 200 mM NaCl, 5 mM imidazole, 2 mM TCEP (added fresh), 5% glycerol, Roche protease inhibitors (1 tablet per 40mL buffer, added on day of use) in warm water, mixing constantly to keep the sample cold. Cells were lysed by two passes through an EmulsiFlex-C3 homog- enizer (Aventin). Protein was extracted from cell membranes by incubation of the crude cell lysate with 1% (w/v) OGNG and 0.1% (w/v) CHS for 1 h at 4 °C on a rotator. Cell debris and unlysed cells were removed by centrifugation at 35,000 g for 45 mins. Immobilized metal affinity chromatography was then used to purify the detergent-solubilized His-tagged protein by batch binding to Co^{2+} charged TALON resin (Clontech) at 4 °C for 1 h. The resin was then washed with wash buffer (WB: 50 mM HEPES (pH 7.5), 5mM

$MgCl_2$, 10 mM imidazole (pH 8.0), 200 mM NaCl , 2 mM TCEP (added fresh), 5% Glycerol, 0.18% OGNG, 0.01 8% CHS, 0.0036% cardiolipin) and the protein was eluted with WB supplemented with 250 mM imidazole (pH 8.0). The eluted protein was desalted using PD-10 columns (GE Healthcare) pre-equilibrated with gel filtration buffer (GFB: 20 mM HEPES (pH 7.5) , 5 mM $MgCl_2$, 200 mM NaCl, 2 mM TCEP, 0.12% OGNG, 0.012% CHS, 0.0024% cardiolipin). Desalted protein was subsequently treated with 10:1 TEV protease (w:w, protein:enzyme) overnight at 4 °C. The TEV protease treated protein was separated from the 6-His-tagged enzymes and uncleaved DPAGT1 by incubation for 1 h with Talon resin (prepared as described above) at 4 °C for 1 h. The resin was collected in a column, the flowthrough collected and the protein sample was centrifuged at 21,500 rpm in a Beckman TA25.5 rotor for 10 min at 4 °C. The supernatant was then concentrated to 0.5 mL using a 30 kDa cutoff PES concentrator (Corning), with mixing every 5 mins during concentration. The concentrated protein was then centrifugated at 20,000 g for 10 min, then further purified by size exclusion chromatography (SEC) on a Sepharose S200 column (GE Healthcare) in GFB. The peak fractions were pooled and concentrated using a Sartorius 2ml PES 50 kDa concentrator (pre-equilibrated with GFB without detergent), at 3220 g. The protein was centrifuged at 20,000 g for 15 mins, then flash frozen in liquid nitrogen. The final concentration was 20-30 mg/mL. Denaturing LC-MS was performed on purified protein obtained from each purification as described below. A mass of 46177 Da was obtained for the WT protein, which matches the DPAGT1 monomer (with an additional N-terminal Serine residue from the TEV cleavage site). Similarly the Val264Gly mutated protein gave a mass of 46135 Da. Each mutated gene was sequenced and the purified protein was subjected to denaturing, intact mass spectrometry. In each case the predicted sequence and mass was observed for the mutated gene and protein. In all cases the monomer mass was observed, and there was no evidence for covalent, disulphide-linked dimers. When DPAGT1 was purified in the absence of reducing agents, the same monomer mass was observed. In no case did we observe a peak with the mass of a covalent, disulphide-bonded dimer.

DPAGT1 Activity Assay

2 μL of 2 μM DPAGT1 WT in GFB buffer (recipe above) supplemented with 5 mM extra $MgCl_2$, 1% OGNG/CHS/cardiolipin and dolichyl monophosphate (100 μM) and tunicamycin

analogue (2 μM) was combined with 2 μL of UDP-N-acetyl [1-^{14}C] D-glucosamine (100 μM) in the same buffer and incubated at 37 °C on a heat block for 21 min. The final concentrations were tunicamycin analogue (1 μM), DPAGT1 (1 μM), dolichyl monophosphate (50 μM), UDP-GlcNAc(1-^{14}C) (50 μM). The reaction was terminated by the addition of 6 μL of 100% methanol and immediately transferred onto ice. 1 μL of sample was spotted onto a silica coated TLC plate in triplicate and run with a mobile phase consisting of chloroform, methanol, and water at a 65:25:4 ratio respectively. After the run, the TLC plate was dried thoroughly, wrapped in cling film, incubated with a phosphor imaging substrate for 4 days, then phosphor imaged using a Biorad. The pixel density of the spots corresponding to the hydrophobic product was divided by combined pixel density of the product and the substrate and multiplied by the known concentration of substrate added to ascertain the amount of product formed. For each compound the assay was repeated for three different biological replicates, and the average and standard deviation were ascertained.

In cell glycoprotein inhibition assays

HEK293T cells were grown to confluency into a standard 12-well culture plate. At this point the media was removed and 250 mL transfection solution was added and the cells were incubated at 37 °C, 5% CO_2 for 10 min. Transfection solution was made up as follows: 20 μL DMEM (serum-free); 69 μL of pHLSec:IgG1Fc-6His plasmid (1152 ng/mL) – 79 μg; 92 mL PEI Max (1 mg/mL) – 92 μg and were mixed and the swirled at room temperature for 20 mins, before addition to the cells.

After 10 min of incubation with the transfection solution, a further 750 mL of DMEM (+2% FBS) was added followed by tunicamycin analogue (3 μL in DMSO – such that the final concentration in the well (i.e. in 1 mL) was 10 mM. 9 further wells were treated under the same conditions with a 3-fold drop in tunicamycin analogue concentration with the tenth well being at 0.5 nM final concentration. The final two wells served as DMSO only controls. The plate was 37 °C, 5% CO_2 for 3 days, before the media was collected and analysed by SDS-page analysis and Western blot. *Note* for Tun-8,2 to achieve higher concentrations the highest concentration used was 5 μL of 60 mM stock in DMSO to give 300 μM, followed by 3 μL of 33 mM to give 100 μM final conc followed by 1/3 dilutions to a final concentration of 15 nM final concentration.

DNA sequence:

Atggggatccttcccagccctgggatgcctgcgctgctctccctcgtgagccttctctccgtgctgctgatgggttgcgtagctgaaac
cggtcacacatgcccaccgtgcccagcacctgaactcctggggggaccgtcagtcttcctcttccccccaaaacccaaggacaccct
catgatctcccggacccctgaggtcacatgcgtggtggtggacgtgagccacgaagaccctgaggtcaagttcaactggtacgtgga
cggcgtggaggtgcataatgccaagacaaagccgcgggaggagcagtacaacagcacgtaccgtgtggtcagcgtcctcaccgtc
ctgcaccaggactggctgaatggcaaggagtacaagtgcaaggtctccaacaaagccctcccagcccccatcgagaaaaccatctc
caaagccaaagggcagccccgagaaccacaggtgtacaccctgcccccatcccgggatgagctgaccaagaaccaggtcagcct
gacctgcctggtcaaaggcttctatcccagcgacatcgccgtggagtgggagagcaatgggcagccggagaacaactacaaggcc
acgcctcccgtgctggactccgacggctccttcttcctctacagcaagctcaccgtggacaagagcaggtggcagcaggggaacgtc
ttctcatgctccgtgatgcatgaggctctgcacaaccactacacgcagaagagcctctccctgtctccgggtggtaccaagcaccacca
tcaccatcact

Protein sequence:

MGILPSPGMPALLSLVSLLSVLLMGCVAETGHTCPPCPAPELLGGPSVFLFPPKPKDT
LMISRTPEVTCVVVDVSHEDPEVKFNWYVDGVEVHNAKTKPREEQYNSTYRVVSVL
TVLHQDWLNGKEYKCKVSNKALPAPIEKTISKAKGQPREPQVYTLPPSRDELTKNQV
SLTCLVKGFYPSDIAVEWESNGQPENNYKATPPVLDSDGSFFLYSKLTVDKSRWQQG
NVFSCSVMHEALHNHYTQKSLSLSPGGTKHHHHHH

SDS Page and Western Blot:

Samples were run on a 4-12% BisTris NuPAGE precast mini-protein gel with MES running buffer. Novex-Sharp Prestained Ladder was added to the first lane followed by the cell-media from the expression tests pre-mixed with Lamelli buffer and heated to 95 °C for 5 mins. Two gels were run for each analogue, with one being stained with Instant-Blue Coomassie stain, and the other being transferred to a nitrocellulose membrane with an iBlot2 mini gel stack transfer device. The membrane was then blocked with 5% BSA in TBS for 2 h, and then incubated at 2 h with a 1:10000 dilution of a primary a-6His antibody conjugated to horseradish peroxidase. The membrane was then washed 4 x TBST, 2 x water and then visualised with the Thermo Pico HRP development kit.

Immunoblotting

To assess the impact of serial dilutions of the tunicamycin analogue Tun-8_E,2_G on the activation of IRE1a in HEK293T cells, 120000 cells per well were seeded in a 12-well plate (Greiner) in DMEM culture medium supplemented with 10% FBS, 2 mM L-Glutamine, 100 U/mL Penicillin – 100 µg/mL Streptomycin and incubated at 37°C, 5% CO_2 for 24 hrs. ER stress was induced by 3-fold serial dilutions of Tun-8_E,2_G analogue (ranging from 100 µM – 5 nM) in reduced serum (0.3%FBS) culture medium at a final 0.3% DMSO concentration. Compound treated, and Low control (0.3% DMSO induced) cells were incubated at 37°C, 5% CO_2 for 6 hrs. For immunodetection cells were lysed in 100 µl modified RIPA-lysis buffer (50mM Tris-HCl pH 7.4, 150nM NaCl, 1% NP-40, 0.25% TritonX-100, 1mM EDTA) supplemented with 80 nM okadaic acid, cOmplete mini EDTA-free protease inhibitor cocktail (Roche) and phosphatase inhibitor cocktail 2 (Merck KGaA). Equal amount of 20mg protein samples were separated in 4–12 % Bis-Tris gels (Invitrogen, Life Technologies) next to PageRuler Plus Prestained Protein Ladder (Thermo Fisher Scientific). Following protein transfer to PVDF membrane with constant 20V for 7 minutes (iBlot2, Life Technologies), the membrane was incubated in blocking buffer (4% BSA) for 1 hr and segmented for antibody probing. After incubation with the primary antibodies phospho-IRE1 alpha (Ser724) (1:500 dilution, Invitrogen, cat. no.: PA1-16927) and beta Actin Loading Control (BA3R) (1:5000 dilution, Invitrogen, cat. no.: MA5-15739) overnight at 4°C, the membrane was washed three times with 1X TBS, 0.1% Tween 20, followed by incubation with HRP-conjugated secondary antibodies Sheep Anti-Rabbit IgG (1:10000 dilution; Merck KGaA, cat. no.: AP5-10P) and Anti-Mouse IgG (H+L) (1:10000 dilution; Promega, cat. no.: WA402B) for 1 hr at room temperature. The proteins were visualised with the SuperSignal West Femto Substrate (ThermoFisher Scientific) on the ChemiDoc MP Imaging System (BioRad Laboratories Inc.). To confirm the phosho-IRE1 signal, the membrane was incubated in Restore Plus Western Blotting Stripping Buffer (Thermo Fisher Scientific) for 5 min followed by blocking with 4% BSA for 1hr. After incubation with primary antibody anti-IRE1(phosphoS724) [EPR5253] (1:10000 dilution; Abcam, cat. no.: ab124945) overnight at 4°C the membrane was washed three times with 1 x TBS, 0.1% Tween 20 (TBS-T), then incubated with HRP-conjugated Anti-Rabbit IgG (H+L) secondary antibody (1:2000 dilution; Promega, cat. no.: AW401B) for 1 hr followed by three washes and then imaged. For non-phosphorylated IRE1 detection the membrane was incubated in Restore Plus Western Blotting Stripping Buffer (1:10000 dilution; Promega, cat. no.: WA402B) for 5 min followed by blocking with 4% BSA for 1hr. After incubation with the primary antibody IRE1a (14C10) (1:1000, Cell Signalling, cat. no.: 3294S) overnight at 4°C the membrane was washed three times with 1X TBS, 0.1% Tween 20, then incubated with

HRP-conjugated Sheep Anti-Rabbit IgG secondary antibody (1:10000 dilution; Merk KGaA, cat. no.: AP5-10P) for 1hr followed by three washes and was imaged.

Supplementary Phenotypic Screening Methods

Method for HEK293.

Tunicamycin analogues were tested in the HEK-ATF4 High Content Imaging assay to assess their ER stress generating ability. Wild-type HEK293 cells were plated in 384-well imaging assay plates at a density of 12,000 cells per well in growth medium (containing DMEM/F12, 10% FBS, 2 mM L-Glutamine, 100 U/mL Penicillin - 100µg/mL Streptomycin) and incubated at 37°C, 5% CO_2. 24-hrs later, the medium was changed to 50 µl assay medium per well (DMEM/F12, 0.3% FBS, 2mM L-Glutamine, 100 U/mL Penicillin – 100 µg/mL Streptomycin). Example compounds were serially diluted in DMSO and then into an intermediate plate using assay medium. Finally, pre-diluted compounds were added to the assay to final concentrations ranging from 10 µM – 5 nM (final DMSO concentration was 0.3%). In addition to the example compound testing area, the plates also contained multiples of control wells for assay normalization purposes, including low control (0.3% DMSO final conc.) and high control (0.3 µM tunicamycin final conc). The assay was started by transferring 5 µl from the intermediate plate into the assay plates, followed by incubation for 6 hrs at 37°C, 5% CO_2. Subsequently, cells were fixed (4% PFA in PBS, 20 min at room temperature followed by washing (3x) with PBS) and submitted to indirect ATF4 immunofluorescence staining. Specifically, cells were treated with primary antibody rabbit anti ATF4 (clone D4B8, Cell Signalling Technologies) in a 1:100 dilution of Perm-block buffer (10% FBS, 0.3% TritonX-100 in PBS) for 2 h, followed by treatment with secondary antibody Alexa Fluor 488 goat anti-rabbit IgG (H+L) (Thermofisher Scientific) in a 1:1000 dilution, and concurrently nuclei staining with Hoechst-33342 dye (Thermofisher Scientific) in 1:400 dilution in Perm-block buffer (5% FBS, 0.3% TritonX-100 in PBS) for 1 h. Plates were imaged on an Opera Phenix High Content imaging platform equipped with 405 nm and 488 nm excitation. Finally, images were analyzed using script-based algorithms. This allowed for determination of the HEK-ATF4 intensity as well as the nuclear:cytoplasmic ratio of ATF4 signal. In addition, HEK-CellCount readout was derived from counting the number of stained nuclei corresponding to healthy cells. This readout served as an internal toxicity control.

Method for Primary Human Dermal Fibroblasts.

A targeted panel of tunicamycin analogues was tested in the ATF4 High Content Imaging assay to assess their ER stress generating ability in a primary cell line. Normal adult human dermal fibroblasts (NHDF-Ad, Lonza) were plated in a 96-well Phenoplate (PerkinElmer) at a seeding density of 10,000 cells per well in fibroblast growth medium (containing fibroblast basal medium, 10% FBS, 0.1% insulin, 0.1% hFGF-B, 0.1% Gentamicin/Amphotericin) and incubated at 37°C, 5% CO2. Treatment with pre-diluted compounds was performed after 24 hrs with final concentrations ranging from 10 μM – 5 nM (final DMSO concentration was 0.3%). Low control (0.3% DMSO final conc.) and high control (0.3 μM tunicamycin final conc.) wells were again included for assay normalization. Treated cells were incubated for 6 hrs at 37°C, 5% CO2 before fixing (4% PFA in PBS, 20 min at room temperature followed by washing (3x) with PBS). Direct ATF4 immunofluorescence staining was then performed, whereby cells were treated with a Coralite Plus 488-conjugated ATF4 Polyclonal antibody (CL488-10835, Proteintech) in a 1:100 dilution of Perm-block buffer (10% FBS, 0.3% TritonX-100 in PBS) for 2 hrs. Nuclei staining was then performed with DRAQ5 dye (ab108410, Abcam) at 1:1000 in PBS. Plates were imaged on a Leica SP8 confocal microscope equipped with 488 nm and 633 nm excitation and images were analysed using script-based algorithms as described for HEK cells above.

Method for ATF4-CHOP Correlation Evaluation in Rat Fibroblasts.

Cell culture: Rat NRK-49F fibroblasts were maintained in culture medium containing DMEM high glucose with GlutaMAX, 10% FBS, 1X Pen-Strep and kept in cell culture incubators at 37 °C; 5% CO_2.

High Content Imaging assay: Cells were seeded into collagen type-1 coated 384-well PE_CCU assay plates (2500 cells/well/ 30 μl). Assay plates were incubated at 37 °C and 5% CO_2 for 48 h. Prior to compound treatment the medium was discarded and 50 μl of fresh assay medium containing DMEM high glucose with GlutaMAX, 0.3% FBS, 1X Pen-Strep was added to the cells. The same treatment medium was used for subsequent addition of tunicamycins, using the same procedures as described for HEK cells and likewise for immunofluorescence processing.

Supplementary Tables

Table S1. % Activity of DPAGT1 in the presence of a tunicamycin analogue compared to a negative control (gel filtration buffer - GFB).

Compound	%ACTIVITY	Stdev
**GFB**	100	10.0
**DMSO**	112.4	10.6
**Tun-9,9**	79.6	2.84
**Tun-i5,9**	90.7	7.39
**Tun-4,9**	93.6	7.24
**Tun-8,8**	90.0	19.2
**Tun-9,5**	54.8	17.0
**Tun-9,4**	83.6	9.02
**Tun-8,A**	35.4	10.1
**Tun**	0.18	0.65

Supplementary Figures

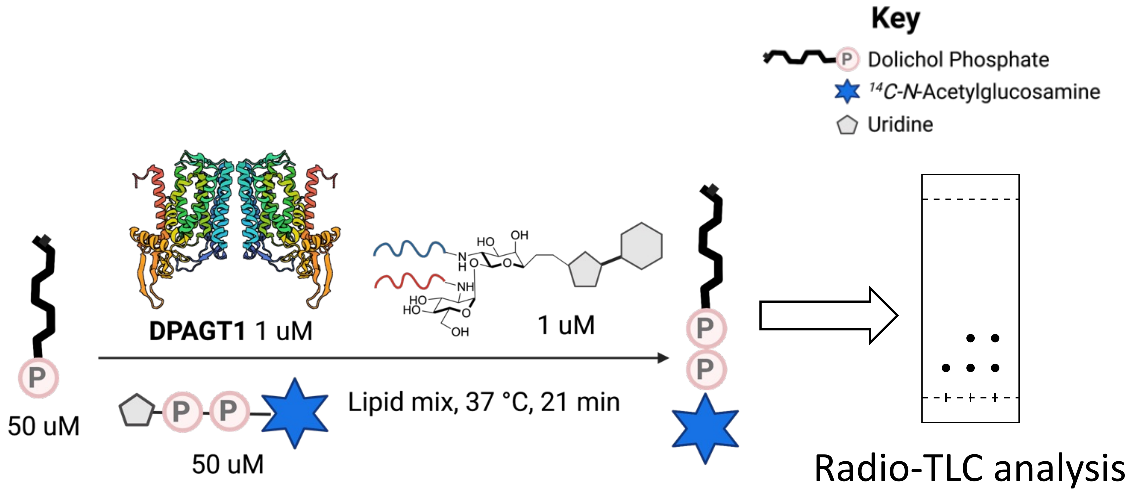

Figure S1. Schematic Representation of the DPAGT1 radiometric assay.

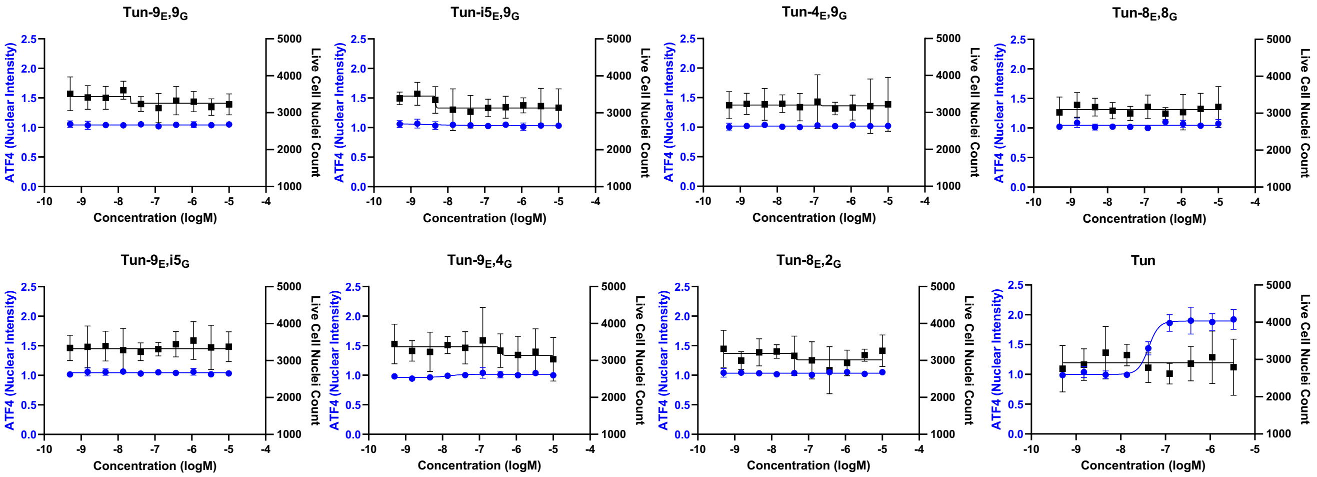

Figure S2. Extracted data from the high-throughput immunofluorescence assay in HEK293 cells depicting nuclear intensity of ATF4 on the left y-axis, and the total live cell count on the right y-axis.

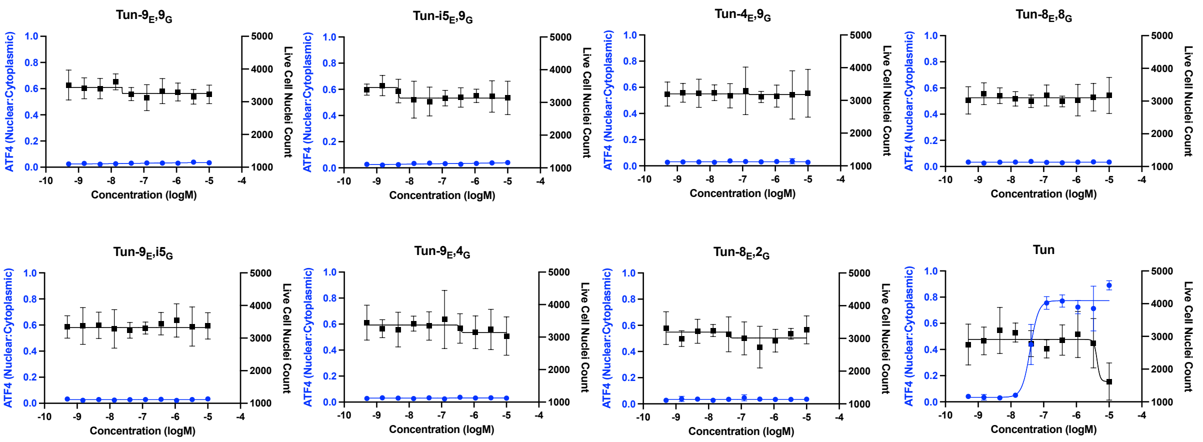

Figure S3. Extracted data from the high throughput immunofluorescence assay in HEK cells depicting nuclear:cytoplasmic ratio of ATF4 on the left y-axis, and the live cell count on the right y-axis.

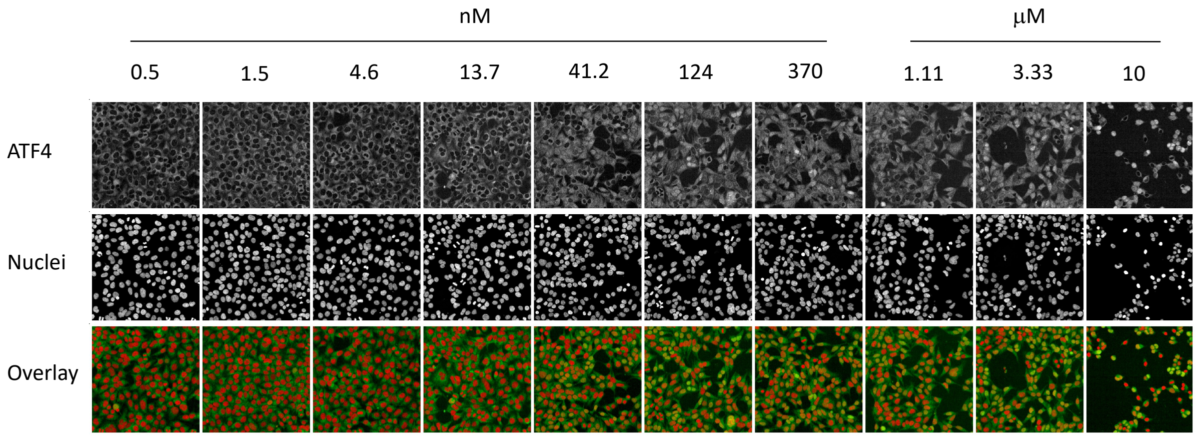

Figure S4. Two channels used in the high-content immunofluorescence assay for tunicamycin in HEK cells.

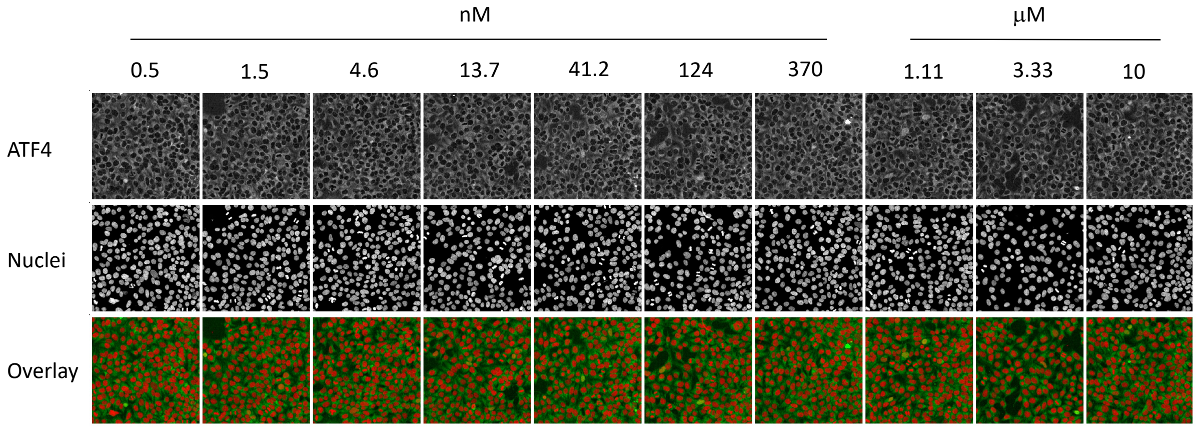

Figure S5. Two channels used in the high-content immunofluorescence assay for Tun-9_E,9_G in HEK cells.

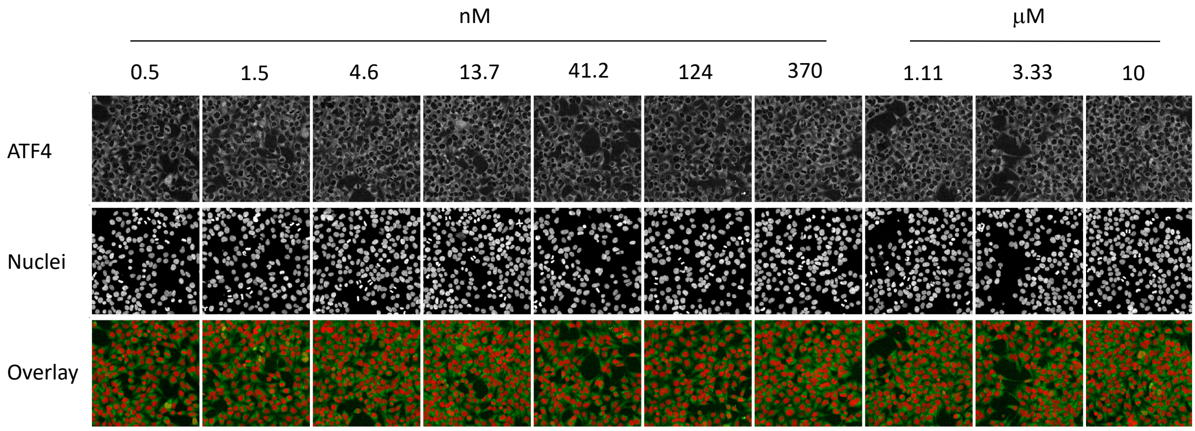

Figure S6. Two channels used in the high-content immunofluorescence assay for Tun-8_E,2_G in HEK cells.

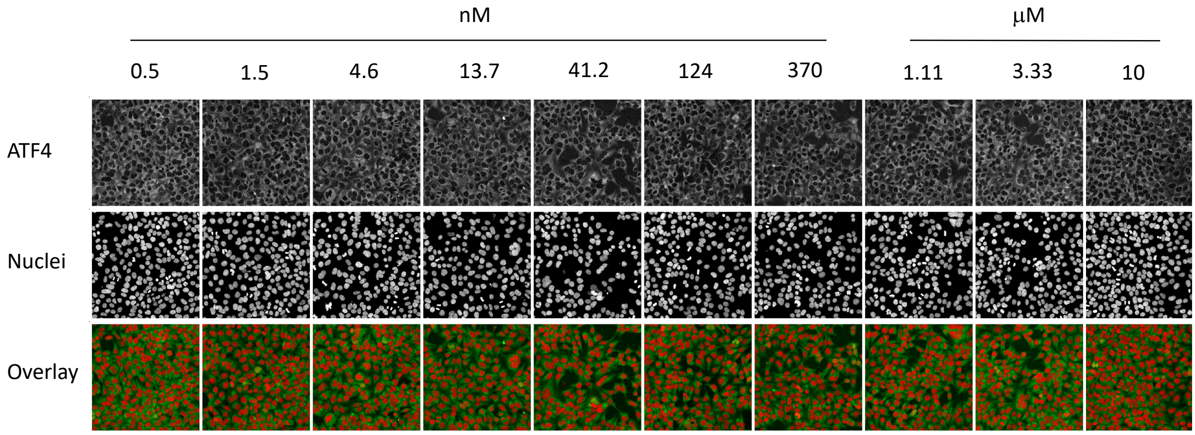

Figure S7. Two channels used in the high-content immunofluorescence for DMSO assay in HEK cells.

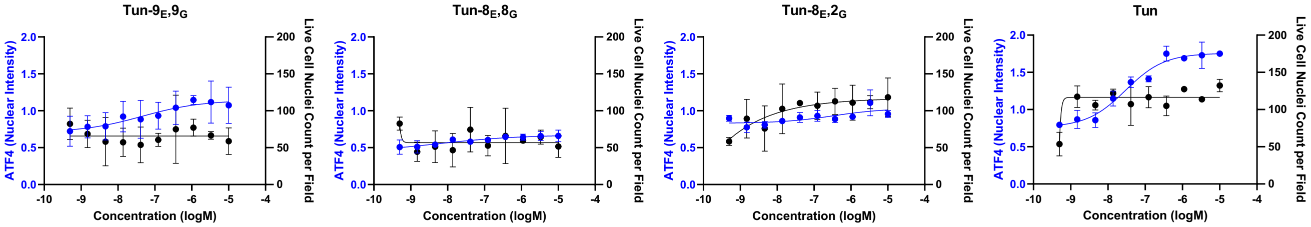

Figure S8. Extracted data from the high-throughput immunofluorescence assay in human dermal fibroblasts depicting absolute nuclear intensity of ATF4 on the left y-axis, and the live cell count per field (14/well) on the right y-axis.

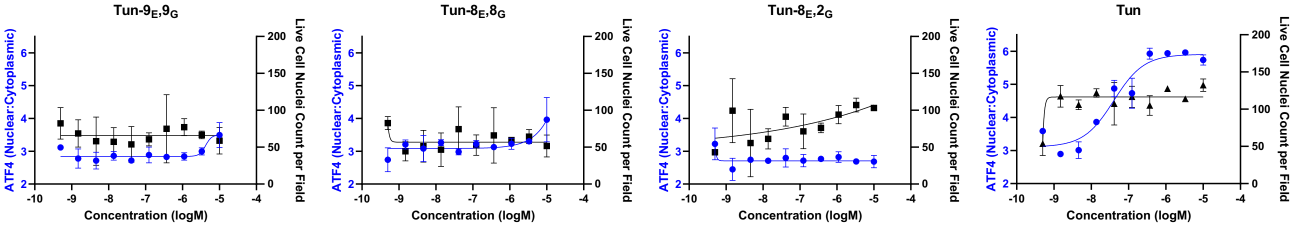

Figure S9. Extracted data from the high-throughput immunofluorescence assay in human dermal fibroblasts depicting nuclear:cytoplasmic ratio of ATF4 on the left y-axis, and the live cell count per field (14/well) on the right y-axis.

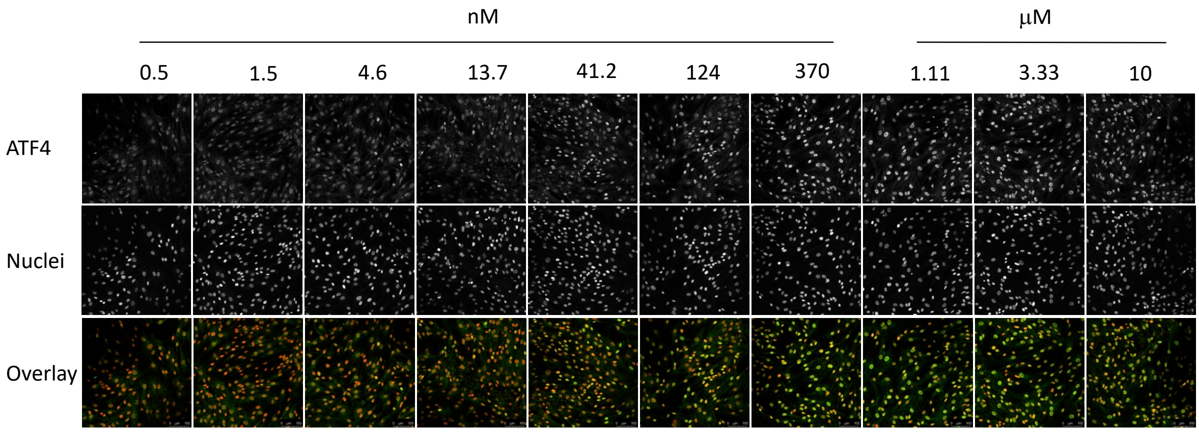

Figure S10. Two channels used in the high-content immunofluorescence assay for tunicamycin in human dermal fibroblasts.

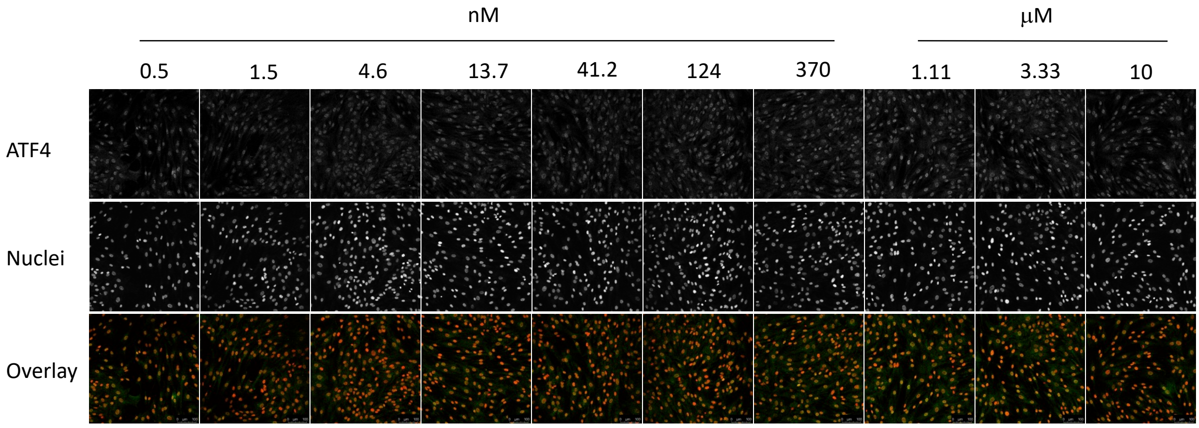

Figure S11. Two channels used in the high-content immunofluorescence assay for Tun-9_E,9_G in human dermal fibroblasts.

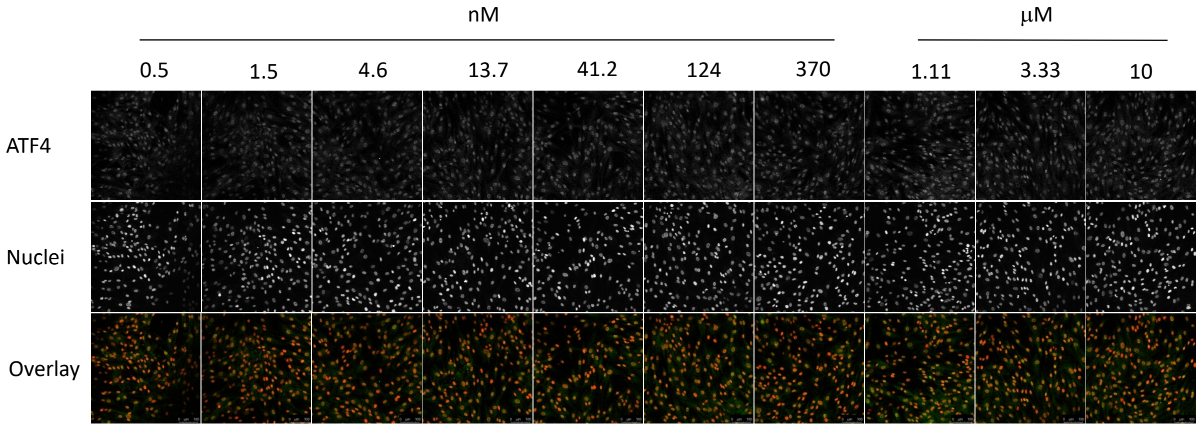

Figure S12. Two channels used in the high-content immunofluorescence assay for Tun-8_E,2_G in human dermal fibroblasts.

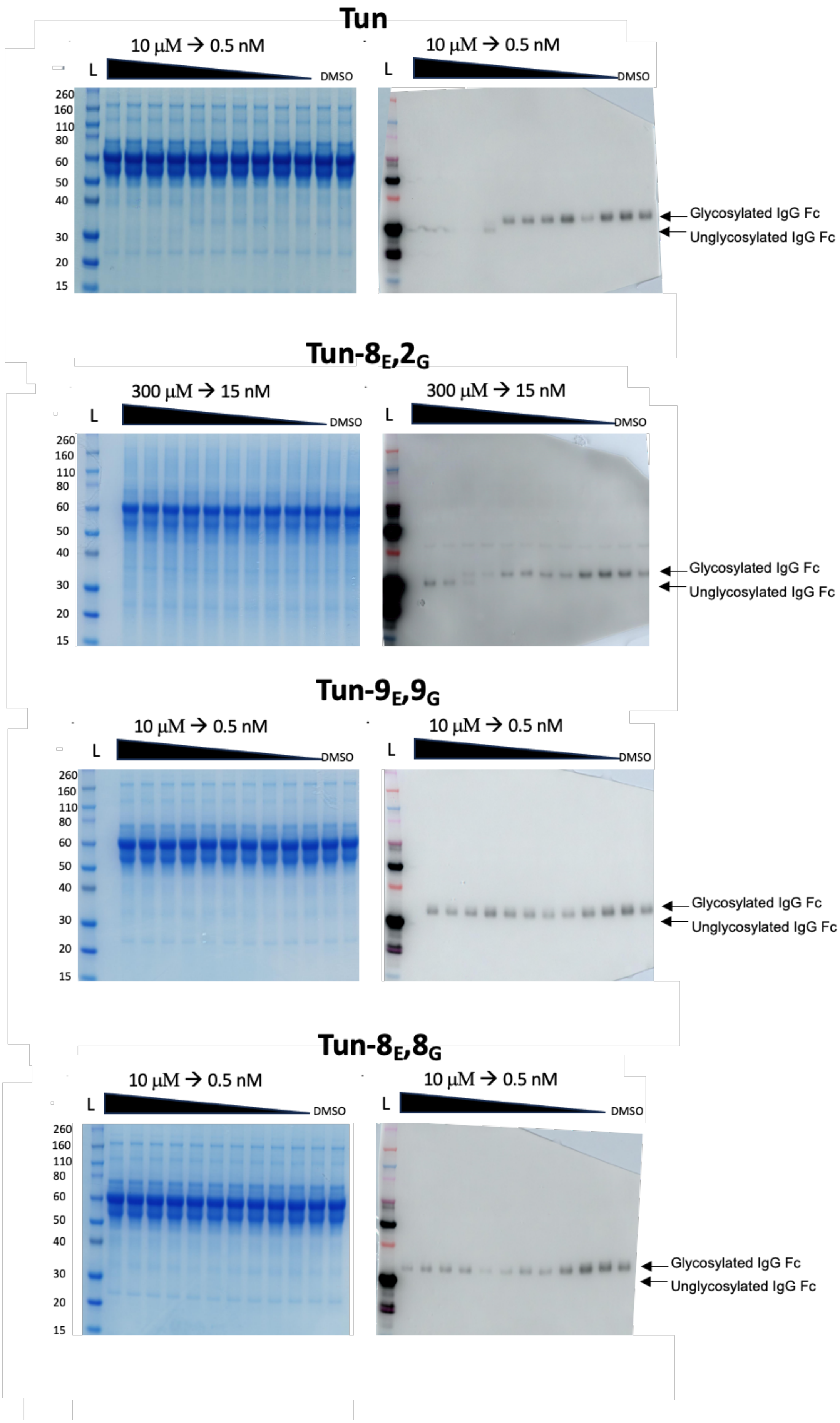
Tun
10 μM → 0.5 nM
L
DMSO
260
160
110
80
60
50
40
30
20
15
Glycosylated IgG Fc
Unglycosylated IgG Fc
Tun-8E,2G
300 μM → 15 nM
Tun-9E,9G
Tun-8E,8G

Figure S13. SDS-Page and Western Blot analysis of a protein glycosylation assay in the presence of four lipid-altered tunicamycin variants.

Each gel has a ladder followed by cell media of HEK293T cells transfected with a plasmid for the expression of an IgG Fc fragment, grown in the presence of 3-fold decreasing concentration of tunicamycin analogue from 10 μM down to 0.5 nM (Tun-8_E,2_G – 300 μM down to 15 nM). The final two lanes in each gel are HEK293T cells grown in the presence of DMSO only. For Western blot analysis the blot was treated with an α-His primary mAb conjugated to HRP and visualised with the Thermo Pico HRP development kit. Consistent with in vitro inhibitory potency, the unglycosylated IgG Fc protein is observed in the presence of tunicamycin and Tun-8_E,2_G with altered windows of concentration variance.

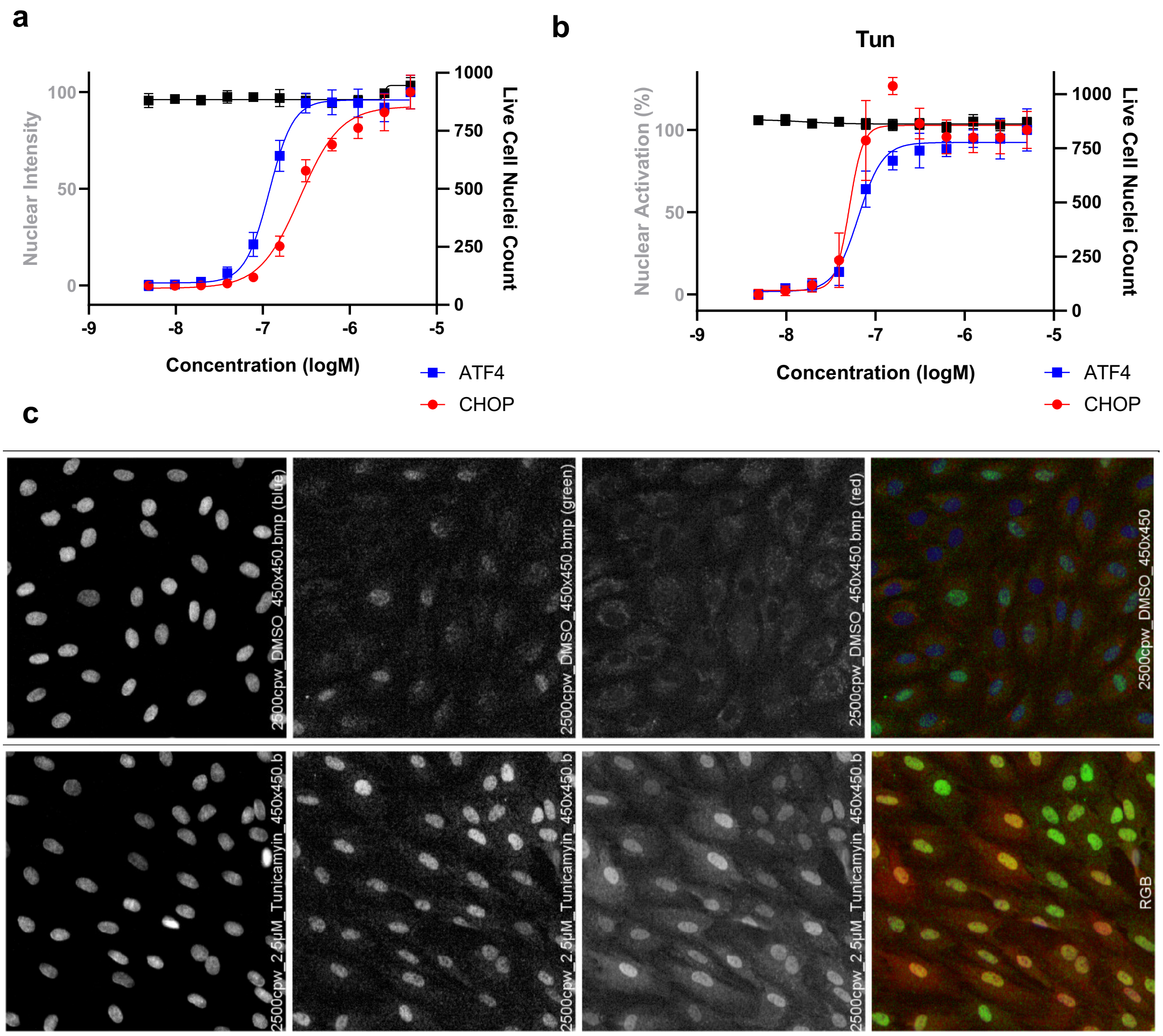

Figure S14. Correlation of CHOP with ATF4 Signal in Rat Fibroblasts.

(**a**) Extracted data from the high-throughput immunofluorescence assay in rat NRK-49F fibroblasts depicting nuclear intensity of ATF4 (blue) and CHOP (red) on the left y-axis, and the live cell count (black) on the right y-axis after 6h of treatment. (**b**) After 18h treatment. (**c**) Representative images of correlated dual CHOP and ATF4 analysis in rat fibroblasts (treatment with 2.5 µM shown).

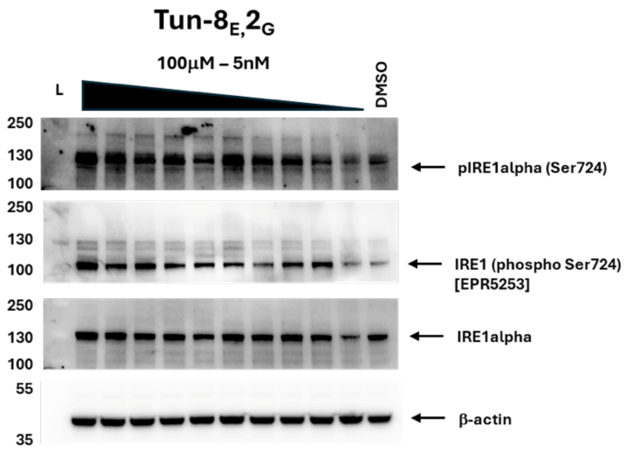

Figure S15. Western Blot analysis of IRE1 phosphorylation response to lipid-altered Tun-8_E,2_G variant.

Levels of phosphorylated and un-phosphorylated IRE1 alpha in wild type HEK293T cells following 6 hours treatment with 3-fold dilutions of the lipid-altered analogue Tun-8_E,2_G from 100 µM to 5 nM. HEK293T cells grown in the presence of 0.3% DMSO are included as negative control. β-actin was used as loading control. Anti-pIRE1alpha PA1-16927 (top) also shows apparent cross-reactivity to IRE1.

Supplementary References

1. Dong, Y. Y., *et al.*, Structures of DPAGT1 Explain Glycosylation Disease Mechanisms and Advance TB Antibiotic Design. *Cell* **2018,** *175* (4), 1045-1058.e16.
